# A functional compartmental model of the *Synechocystis* PCC 6803 phycobilisome

**DOI:** 10.1007/s11120-017-0424-5

**Published:** 2017-07-18

**Authors:** Ivo H. M. van Stokkum, Michal Gwizdala, Lijin Tian, Joris J. Snellenburg, Rienk van Grondelle, Herbert van Amerongen, Rudi Berera

**Affiliations:** 1 Faculty of Sciences, Institute for Lasers, Life and Biophotonics, VU University Amsterdam, De Boelelaan 1081, 1081 HV Amsterdam, The Netherlands; 2 Department of Physics, University of Pretoria, Pretoria, South Africa; 3 Laboratory of Biophysics, Wageningen University, Wageningen, The Netherlands; 4 Department of Food Sciences, Faculty of Agriculture, Kagawa University, Miki-cho, Kagawa 761-0795 Japan

**Keywords:** Excitation energy transfer, Global analysis, Light harvesting, Orange carotenoid protein, Target analysis

## Abstract

In the light-harvesting antenna of the *Synechocystis* PCC 6803 phycobilisome (PB), the core consists of three cylinders, each composed of four disks, whereas each of the six rods consists of up to three hexamers (Arteni et al., Biochim Biophys Acta 1787(4):272–279, 2009). The rods and core contain phycocyanin and allophycocyanin pigments, respectively. Together these pigments absorb light between 400 and 650 nm. Time-resolved difference absorption spectra from wild-type PB and rod mutants have been measured in different quenching and annihilation conditions. Based upon a global analysis of these data and of published time-resolved emission spectra, a functional compartmental model of the phycobilisome is proposed. The model describes all experiments with a common set of parameters. Three annihilation time constants are estimated, 3, 25, and 147 ps, which represent, respectively, intradisk, interdisk/intracylinder, and intercylinder annihilation. The species-associated difference absorption and emission spectra of two phycocyanin and two allophycocyanin pigments are consistently estimated, as well as all the excitation energy transfer rates. Thus, the wild-type PB containing 396 pigments can be described by a functional compartmental model of 22 compartments. When the interhexamer equilibration within a rod is not taken into account, this can be further simplified to ten compartments, which is the minimal model. In this model, the slowest excitation energy transfer rates are between the core cylinders (time constants 115–145 ps), and between the rods and the core (time constants 68–115 ps).

**Electronic supplementary material:**

The online version of this article (doi:10.1007/s11120-017-0424-5) contains supplementary material, which is available to authorized users.

## Introduction

Photosynthesis is key to the conversion of solar energy to biomass. Light-harvesting antennae absorb sunlight and transfer the excitation energy ultimately to the reaction centers. The phycobilisome (PB) is the light-harvesting antenna of many cyanobacteria, red algae, and glaucophytes (Adir 2005; Glazer 1984; Watanabe and Ikeuchi 2013). In cyanobacteria, its shape is hemidiscoidal, with a central core from which 4–8 rods radiate. In *Synechocystis* PCC 6803 (hereafter *Synechocystis*), the core consists of three cylinders, each composed of four disks, whereas each of the six rods consists of up to three hexamers (Arteni et al. 2009). Light is absorbed by phycocyanobilin pigments that are covalently bound to phycobiliproteins (Glazer 1984). The rods and core contain phycocyanin (PC) and allophycocyanin (APC), respectively. Together these pigments absorb light between 400 and 650 nm, and excitations of the antenna pigments are efficiently transferred to the chlorophyll-containing photosystems I and II (Tian et al. 2011, 2012, 2013b; Scott et al. 2006; Gillbro et al. 1985; Sandstrom et al. 1988; Liu et al. 2013). These photosystems convert the excitations to chemical energy via initial charge separation (van Grondelle et al. 1994), and the combined action of PBs and photosystems (the light reactions of photosynthesis) provides the energy input to the cell. The kinetics of excitation energy transfer (EET) in the PB, in particular the microscopic rates describing the coupling within the rods, between rods and core, within the core, and between the core and photosystems I and II, are only partly known (Tian et al. 2011; Holzwarth 1991). To describe the light-harvesting function of the PB a model is needed, which quantifies the dynamics and the distribution of all absorbed light energy to photosystems I and II.

Recently, using time-resolved fluorescence spectroscopy, we have investigated intact *Synechocystis* cells and isolated PBs (both wild-type and mutants) (Tian et al. 2011, 2012, 2013a, b). Although the signal-to-noise ratio (SNR) was excellent, the time resolution of these experiments (≈10 ps) was not sufficient to resolve the fastest EET components. Here, we employ time-resolved difference absorption spectroscopy (Berera et al. 2009), with a time resolution of ≈0.2 ps. The down side of these ultrafast measurements is the lower SNR. When applying more laser power in order to increase this SNR, complications arise due to the absorption of more than one photon by a PB. Multiple excitations will annihilate, until a single excitation remains. This is a blessing in disguise, since these annihilation processes also provide information on the dynamics within the PB (Bakker et al. 1983).

**Fig. 1:**
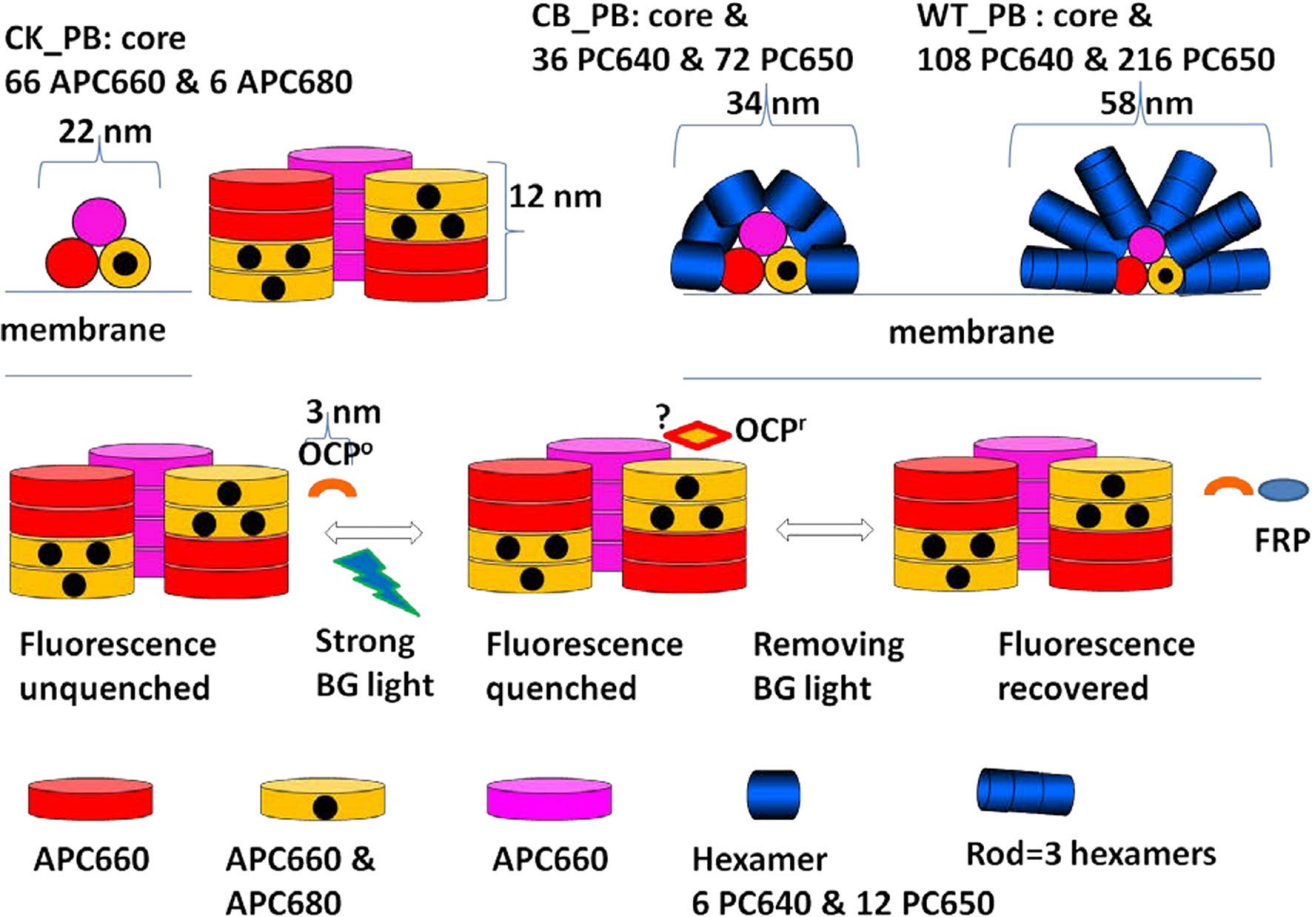
The structure of each type of PB is shown schematically: CK has no rods, in CB each of the six rods contains one hexamer, and in WT the rods contain three hexamers. PC rods in *blue* (number of PC640 and PC650 pigments indicated). APC that fluoresces at 660 nm (APC660) in *magenta*, *red* and* orange* (66 pigments in total), and the low-energy part of APC indicated by *black dots* (six APC680 pigments in total). The approximate length for each subunit is based on (Arteni et al. 2009). The question mark (‘‘*?*’’) indicates the potential pigments that OCPr is interacting with. Linker proteins have been omitted for clarity

Based upon structural data (Arteni et al. 2009), a model has been proposed for the pigment organization in the *Synechocystis* PB (Fig. 1). The core consists of two basal and a top cylinder. It possesses a C2 rotational symmetry axis, perpendicular to the membrane. Each cylinder consists of a stack of four disks. Each disk contains six APC pigments (Reuter et al. 1999; Figure S1), in the top cylinder only APC660 (660 nm is the wavelength of maximal emission). In half of the basal disks, one or two APC680 pigments are present (these are called the D and EF disk after the relevant genes, respectively). The APC680 pigments are the so-called terminal emitters (TE), indicated by black dots. The APC660 pigments in the D and EF disk that equilibrate quickly with the APC680 pigments in their disk are indicated by orange. The remaining APC660 pigments in the basal cylinders are indicated by red, whereas the APC660 pigments in the top cylinder are indicated by magenta. In the PBs from the CK mutant rods are absent (Piven et al. 2005), whereas in the CB mutant (Ughy and Ajlani 2004), a rod consists of a single hexamer. Each PC hexamer contains 18 pigments (indicated blue), six PC640 on the outside, and 12 PC650 in the center. In the WT PB each rod consists of three hexamers on average, and thus this pigment protein complex contains up to 108 PC640, 216 PC650, 66 APC660, and six APC680 pigments, in total 396 pigments. Every pigment is covalently bound to a phycobiliprotein, and together with the colorless linker proteins the whole pigment protein complex is built up from 318 pigment-proteins.

When illuminated by strong blue green light, the PB is subjected to fast, reversible energy-dependent quenching. The major photoprotective energy dissipation mechanism is mediated by an orange carotenoid protein (OCP) (Wilson et al. 2006), which upon photoactivation docks on the PB (Gwizdala et al. 2011). The carotenoid in OCP, the 3′-hydroechinenone, when excited converts its orange form (OCP^o^) to a red form (OCP^r^) (Wilson et al. 2008), that interacts with the PB core, thereby inducing the quenching of the PB excitations at the level of APC660 (Tian et al. 2011, 2012; Jallet et al. 2012). The quenching can be turned off by a fluorescence recovery protein (FRP) (Boulay et al. 2010). The precise site of interaction between OCP^r^ and PB is being debated, as is the precise molecular mechanism (Kirilovsky 2015; Stadnichuk et al. 2015; Zhang et al. 2014). Recently, a different light-induced quenching mechanism signified by the fluorescence blinking in single molecule spectroscopy measurements has been demonstrated in individual PB complexes as well as in isolated rods and cores of PB (Gwizdala et al. 2016).

In this paper, a functional compartmental model of the PB will be developed. A compartment contains pigments with the same light-harvesting function that are considered equivalent. The four colors that are used in Fig. 1 for the CK core, magenta, red, orange, and black, indicate the four groups of pigments that will be lumped into four compartments. Microscopic rate constants describe the rates of EET between the compartments. The spectral properties of the different compartments will be described by species-associated (difference) spectra. The parameters of this target model will be estimated from the time-resolved emission and difference absorption spectra. The novel difference absorption experiments of CK, CB, and WT PB in vitro will be described in detail. The emission experiments of CK, CB, and WT PB in vitro from (Tian et al. 2012) will be reanalyzed. Below we will first globally analyze all the data and based thereupon develop a unified functional compartmental model describing all these experiments with a common set of parameters.

## Methods

### Sample preparation

PB from WT *Synechocystis* and from the CK (Piven et al. 2005) and CB (Ughy and Ajlani 2004) mutants of *Synechocystis* were isolated as described previously (Gwizdala et al. 2011). Samples were stored at −80 °C. OCP was isolated as reported before in Wilson et al. (2008) from the overexpressing C-terminal His-tagged OCP—ΔCrtR *Synechocystis* (Wilson et al. 2011). Quenched OCP-PB complexes were prepared like in Gwizdala et al. (2011).

### Time-resolved difference absorption spectroscopy measurements

Transient absorption spectroscopy was performed on a Coherent MIRA seed and RegA amplifier system. The initial pulse at 800 nm and ≈60 fs full width at half maximum (FWHM) is split into two beams. One is used to pump an optical parametric amplifier to generate the pump beam with ≈100 fs bandwidth. The excitation wavelength was ≈622 nm (WT PB, selectively exciting the rods), ≈650 nm (CB PB, mainly exciting the core) and ≈656 nm (CK PB). The beam size was ≈160 μm. The pulse energy was <0.5, 2, 3, and 5 nJ for unquenched CB PB and 2, 3, and 5 nJ for quenched CB PB-OCP. It was 2, 8, and 12 nJ for unquenched CK PB, and 2, 5, and 10 nJ for unquenched WT. The second beam is focused on a CaF_2_ plate to generate a white light continuum, the probe beam. The repetition rate was set to 40 kHz and the polarization between the pump and the probe beam set at the magic angle (54.7°). The detection system with CB PB consists of a 75 diode array (587–717 nm) coupled to a home built shot-to-shot detector. This new detection system allows faster measuring times because we can detect more wavelengths simultaneously and the SNR is higher since it allows for a better control of the quality of the data (acquisition vs rejection). A 256 diode array (450–750 nm) with lower SNR was used with CK PB and WT PB. Finally, an extensive power series of the unquenched CB PB was measured using an excitation wavelength of ≈671 nm (mainly exciting the core) and powers of 10, 5, 3, 2, 1, and 0.5 nJ. Here, a 15 diode array was used, in which 15 wavelengths between 645 and 700 nm were measured with a very high SNR.

### Time-resolved emission measurements

Time-resolved emission spectra of the CK PB, CB PB, and WT PB in vitro have been described in (Tian et al. 2012). The excitation wavelength was ≈590 nm (mainly exciting the rods in CB PB and WT PB), and both PB unquenched and PB quenched by OCP^r^ were measured. The total time range was 2 ns, with an instrument response function (IRF) of ≈22 ps FWHM. In addition, WT PBs were also measured with a total time range of 800 ps, with an IRF of ≈10 ps FWHM.

### Global and target analysis of time-resolved spectra

In target analysis of time-resolved difference absorption spectra, the inverse problem is to determine the number of electronically excited states (${N_{{states}}}$) present in the system, and to estimate their spectral properties ${\text{SADS}}_{l} (\lambda )$ (species-associated difference spectra) and their populations $c_{l}^{S}(t)$(superscript S stands for species). The time-resolved spectra $\text{TRS}(t,\lambda )$ are described by a parameterized superposition model:

$${\text{TRS}}(t,\lambda ) = \sum\nolimits_{{l = 1}}^{{N_{{{\text{states}}}} }} {c_{l}^{S} (t,\theta ){\text{SADS}}_{l} (\lambda )},$$

where the populations are determined by an unknown compartmental model that depends upon the unknown kinetic parameters $\theta$. In the target analysis, constraints on the *SADS* are needed to estimate all parameters $\theta$ and ${\text{SADS}}_{l} (\lambda ).$ In order to also describe the vibrational evolution and the coherent artefact we add a superposition of damped oscillations (van Stokkum et al. 2016). The amplitude of a damped oscillation $\cos ({\omega _n}t)\exp ( - {\gamma _n}t)$ as a function of the detection wavelength constitutes a damped oscillation-associated spectrum ${\text{DOAS}}_{n} (\lambda )$ with an accompanying phase characteristic ${\varphi _n}(\lambda )$. Thus, we arrive at the model function:

$${\text{TRS}}(t,\lambda ) = \sum\nolimits_{{l = 1}}^{{N_{{{\text{states}}}} }} {c_{l}^{S} (t^{\prime},\theta ){\text{SADS}}_{l} (\lambda )} + \sum\nolimits_{{n = 1}}^{{Nosc}} {{\text{DOAS}}_{n} (\lambda )\cos (\omega _{n} t^{\prime} - } \varphi _{n} (\lambda ))\exp ( - \gamma _{n} t^{\prime}),$$

where $t'$ indicates that the actual model function still has to take into account the IRF (*vide infra*). The population of the *l*-th compartment is $c_{l}^{S}(t)$. The concentrations of all compartments are collated in a vector: $${c^S}(t)={\left[ {\begin{array}{*{20}{c}} {c_{1}^{S}(t)}&{c_{2}^{S}(t)}& \ldots &{c_{{{n_{comp}}}}^{S}(t)} \end{array}} \right]^{T}}$$ which obeys the differential equation

$$\frac{d}{{dt}}{c^S}(t)=K{c^S}(t)+j(t),$$

where the transfer matrix *K* contains off-diagonal elements ${k_{pq}}$, representing the microscopic EET rate constant from compartment *q* to compartment *p*. The diagonal elements contain the total decay rates of each compartment. The input to the compartments is $$j(t) = {\text{IRF}}(t)\left[ {x_{1} \begin{array}{*{20}c} \ldots & {x_{{n_{{comp}} }} } \\ \end{array} } \right]^{T}$$, with ${x_l}$ the absorption of the *l*-th compartment.

The impulse response of the system, which is a sum of exponential decays, has to be convolved with the IRF. Typically, a Gaussian shaped IRF is adequate, with parameters* μ* for the location of the IRF maximum and Δ for the FWHM of the IRF:

$${\text{IRF}}(t) = \frac{1}{{\tilde{\Delta }\sqrt {2\pi } }}\exp ( - \log (2)(2(t - \mu )/\Delta )^{2} )$$

where $\tilde{\Delta } = \Delta /\left( {2\sqrt {2\log (2)} } \right)$. The convolution (indicated by an *) of this IRF with an exponential decay (with decay rate *k*) yields an analytical expression which facilitates the estimation of the decay rate *k* and the IRF parameters *µ* and Δ:

$$c_{{}}^{D} (t,k,\mu ,\Delta ) = \exp ( - kt) * {\text{IRF}}(t) = \frac{1}{2}\exp ( - kt)\exp \left( {k\left( {\mu + \frac{{k\tilde{\Delta }^{2} }}{2}} \right)} \right)\left\{ {1 + {\text{erf}}\left( {\frac{{t - (\mu + k\tilde{\Delta }^{2} )}}{{\sqrt 2 \tilde{\Delta }}}} \right)} \right\}.$$

When the compartmental model consists of independently decaying species, with populations $c_{l}^{D}(t,{k_l},\mu ,\Delta )$ (superscript D stands for decay) their spectra are termed ${\text{DADS}}_{l} (\lambda )$ (decay-associated difference spectra). The solution of the general compartmental model described by the *K* matrix consists of exponential decays with decay rates equal to the eigenvalues of the *K* matrix. The interrelation between the DADS and SADS is expressed in the following matrix equation:

$$C^{D} (\theta ,\mu ,\Delta ) \cdot {\text{DADS}}^{{\text{T}}} = C^{S} (\theta ,\mu ,\Delta ) \cdot {\text{SADS}}^{{\text{T}}}$$

Here the matrix ${C^D}(\theta ,\mu ,\Delta )$ contains in its *l*-th column the decay $c_{l}^{D}(t,{k_l},\mu ,\Delta )$ and the matrix ${C^S}(\theta ,\mu ,\Delta )$ contains in its columns the populations $c_{l}^{S}(t)$ of the general compartmental model. The estimation of the DOAS is detailed in (van Stokkum et al. 2016). When the compartmental model consists of a sequential scheme with increasing lifetimes the spectra are termed ${\text{EADS}}_{l} (\lambda )$ (evolution-associated difference spectra).

In target analysis, also the time-resolved emission spectra $\text{TRES}(t,\lambda )$ are described by a parameterized superposition model:

$${\text{TRES}}(t,\lambda ) = \sum\limits_{{l = 1}}^{{N_{{{\text{states}}}} }} {c_{l}^{S} (t^{\prime } ,\theta ){\text{SAS}}_{l} (\lambda )}$$

with ${\text{SAS}}_{l}(\lambda )$ the species-associated spectra. When the compartmental model consists of independently decaying species their spectra are termed ${\text{DAS}}_{l}(\lambda )$ (decay-associated spectra), and when it consists of a sequential scheme with increasing lifetimes the spectra are termed ${\text{EAS}}_{l}(\lambda )$ (evolution-associated spectra). Typically, the IRF can be well approximated by a sum of up to three Gaussians.

### Simultaneous target analysis

To resolve the different species and to improve the precision of the estimated parameters, the set of ${N_{\exp }}$ experiments that describe the same sample (measured in different quenching or annihilation conditions) can be analyzed simultaneously. For each additional data set ${\text{TRS}}_{e}$ one scaling parameter ${\alpha _e}$ and one time shift parameter ${\mu _e}$ must be added:

$${\text{TRS}}_{e} = \alpha _{e} (C_{e} ^{S} (\theta ,\mu _{e} ,\Delta )) \cdot {\text{SADS}}^{{\text{T}}}$$

The scaling parameter ${\alpha _e}$ thus describes the different excitation power used. The extensions of the basic EET model which are needed to describe the measurements in the different quenching or annihilation conditions will be described below.

### Residual analysis

Following a successfully converged fit, the matrix of residuals is analyzed with the help of a singular value decomposition (SVD). Formally the residual matrix can be decomposed as

$$\text{res}(t,\lambda )=\sum\limits_{l=1}^m {u_{{_{l}}}^{{res}}(t){s_l}w_{{_{l}}}^{{res}}(\lambda )}$$

where ${u_l}$ and ${w_l}$ are the left and right singular vectors; ${s_l}$ is the sorted singular values, and *m* is the minimum of the number of rows and columns of the matrix. The singular vectors are orthogonal, and provide an optimal least squares approximation of the matrix. The SVD of the matrix of residuals is useful to diagnose shortcomings of the model used, or systematic errors in the data.

## Results and discussion

### Time-resolved difference absorption spectroscopy (TRS)

#### Unquenched phycobilisome core (CK PB)

**Fig. 2:**
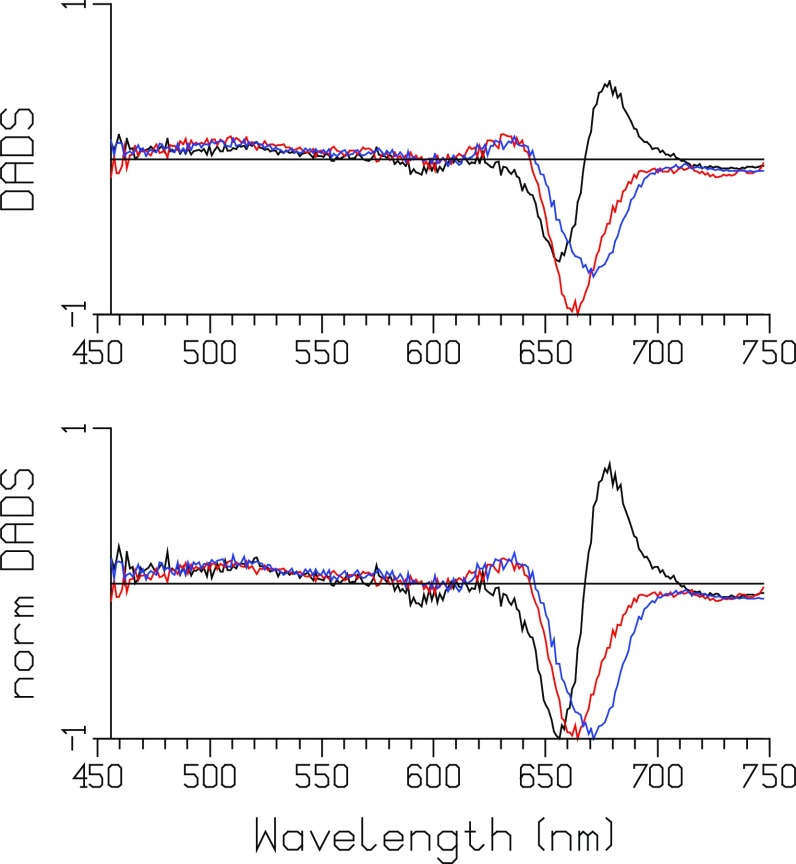
Decay-associated difference spectra (DADS) of CK PB after 12 nJ excitation at 656 nm. Lifetimes (color key): 9 ps (*black*), 75 ps (*red*), 1.4 ns (*blue*). At the bottom normalized DADS. The DOAS are not shown

The decay-associated difference spectra (DADS) depicted in Fig. 2 show equilibration and EET within the core after 12 nJ excitation at ≈656 nm. Concomitantly, annihilation processes take place in those complexes absorbing two or more photons. Sub ps phenomena (described by DOAS) will be discussed below. The first DADS (9 ps, black) represents the EET from APC660 to APC680. It is almost conservative (i.e., the negative and positive areas nearly cancel), indicating that little annihilation is present during the 9 ps time window. After the 9 ps equilibration, the red DADS represents a loss of bleach plus SE, evidencing annihilation that takes place with a 75 ps time constant. This indicates that the final equilibration of the phycobilisome core (CK) takes ≈75 ps. The blue DADS, decaying in 1.4 ns, represents the terminal emitter APC680 (six pigments) in equilibrium with APC660 (66 pigments). Its bleach plus stimulated emission (SE) minimum is at ≈672 nm. The 75 ps DADS (red) is blue shifted relative to the 1.4 ns DADS (blue), indicating that during the annihilation relatively more APC660 bleach plus SE disappear.

Measurements at lower powers of 8 and 2 nJ show similar equilibration, but less annihilation than in Fig. 2 (see Figure S2).

### Unquenched phycobilisome core with short rods (CB PB)

**Fig. 3:**
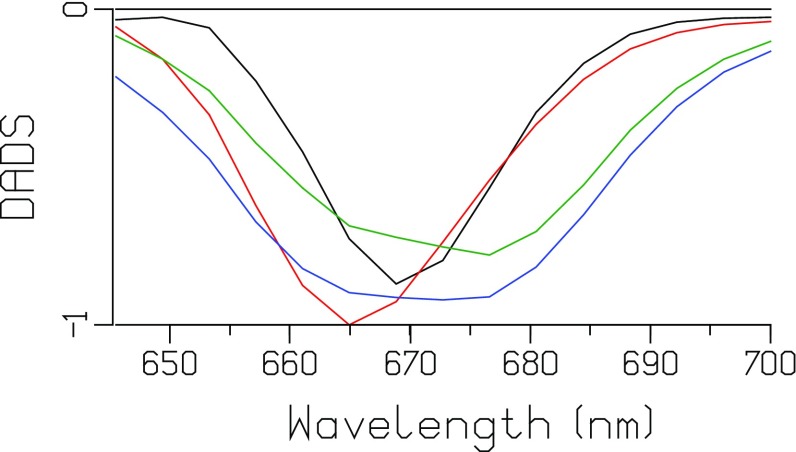
DADS of CB PB after 10 nJ excitation at 671 nm. Lifetimes (color key): 2.5 ps (*black*), 19 ps (*red*), 138 ps (*blue*), 1.5 ns (*green*)

An extensive power series of the unquenched CB particle was measured using an excitation wavelength of ≈671 nm and powers from 10 down to 0.5 nJ. At the highest power of 10 nJ (Fig. 3) relatively more annihilation takes place than in Fig. 2. The final DADS (1.5 ns, green) represents the terminal emitter APC680 (six pigments) in equilibrium with APC660 (66 pigments) and the six rods (each 6 PC640 and 12 PC650). Its bleach plus SE minimum is at ≈676 nm. The other three DADS are of similar magnitude, indicating a net loss of bleach plus SE with time constants of 2.5, 19, and 138 ps. Since the final DADS (1.5 ns, green) represents one excitation, the similar areas of the first three DADS suggest that in each of these DADS one excitation gets lost due to annihilation. A monotonic increase in the loss of bleach plus SE with increasing power is visible in the series of DADS depicted in Figure S3. These timescales can loosely be interpreted as intradisk (1.6–2.6 ps), interdisk/intracylinder (13–19 ps), and intercylinder (131–163 ps) equilibration and annihilation.

**Fig. 4:**
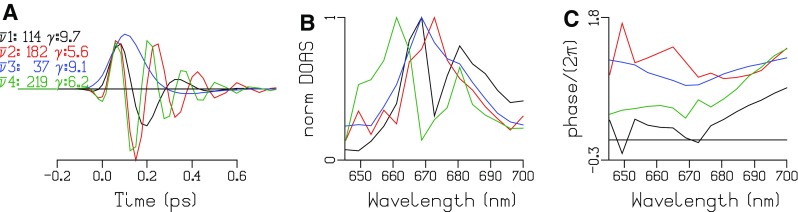
**a** Cosine oscillations with frequencies $\overline {\nu \,} n$ (in /cm) (where n is the DOAS number) and damping rates *γ* (in 1/ps) written in the legend at the *left*, using the appropriate color. **b** Estimated DOAS (normalized, with number indicated in the legend at the far *left*). **c** Estimated phase profiles of the DOAS

The sub ps phenomena can be described by four DOAS (Fig. 4). The FWHM of the IRF is 138 fs, and the decay rates of the DOAS vary between 5 and 10/ps. The dominant DOAS (37/cm, blue) peaks at the excitation wavelength and is attributed to a coherent artefact. The higher frequencies (114/cm, black, 182/cm, red and 219/cm, green) correspond to the dominant oscillation around 200/cm resolved in an APC trimer (Zhang et al. 2001). The relatively large IRF width and the step size of 25 fs preclude resolving higher frequencies.

### Quenched and unquenched phycobilisome core with short rods (CB PB)

The spectral evolution of quenched and unquenched CB particles is demonstrated in Figure S4. The lifetimes of the unquenched sample in Table S3 agree with those in Table S2. In addition to the loss of bleach plus SE area due to annihilation, an additional loss due to the OCP-related energy quenching is clearly visible, comparing the light-colored and dark-colored evolution-associated difference spectra (EADS) in Figure S4. Only part of the sample was quenched, which complicates the interpretation of the lifetimes shortened by both energy quenching and annihilation.

### Unquenched phycobilisome (WT PB)

**Fig. 5:**
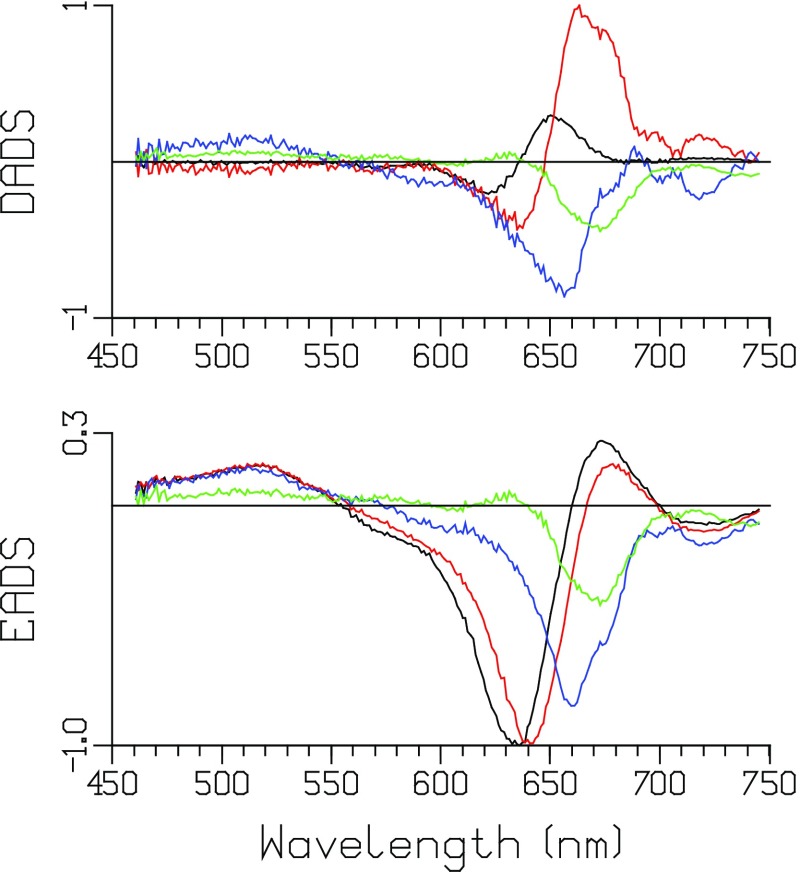
DADS and EADS of WT PB after 5 nJ excitation at 622 nm. Lifetimes (color key): 5 ps (*black*), 59 ps (*red*), 132 ps (*blue*), 1.2 ns (*green*). The DOAS are not shown

The DADS and EADS depicted in Fig. 5 show equilibration and EET within the wild-type phycobilisome after 5 nJ excitation at ≈622 nm. The initial excitation selects mainly the PC640 pigments, which equilibrate in ≈5 ps with the PC650 pigments. Then in 59 ps the rods equilibrate with the core. Equilibration of the PB is complete in 132 ps. Concomitantly, annihilation processes take place in those complexes absorbing two or more photons. The bleach plus SE area of the blue EADS is smaller than that of the red EADS, indicating some annihilation with a 59 ps time constant. The blue DADS indicates a loss of bleach plus SE, evidencing annihilation with a 132 ps time constant.

Measurements at powers of 2 and 10 nJ show similar equilibration, but less and more annihilation than in Fig. 5, respectively (cf. Figure S5).

### Time-resolved emission spectroscopy (TRES)

#### Global analysis of unquenched and quenched TRES of CK PB

**Fig. 6:**
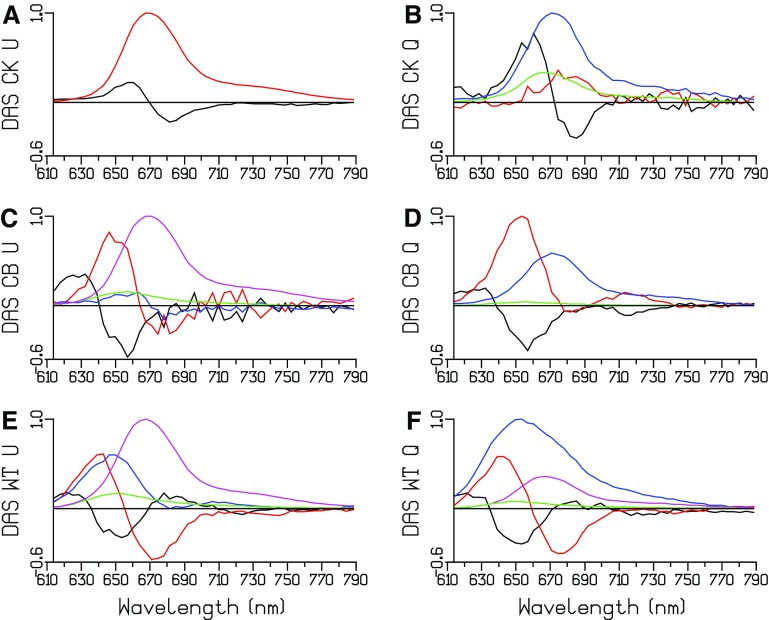
Estimated DAS of unquenched and quenched CK, CB, and WT PB after 590 nm excitation. Estimated lifetimes are collated in Table 1

The TRES data from (Tian et al. 2012) have been simultaneously reanalyzed. In Fig. 6a, b, the DAS estimated from the unquenched and quenched TRES of CK is depicted. Because of the 24 ps FWHM of the IRF, it is very difficult to estimate the fastest equilibration time constants. An average equilibration time of 43 ps is estimated in unquenched CK, with a conservative DAS (black in Fig. 6a). In quenched CK, the first lifetime speeds up to 25 ps, and the DAS is no longer conservative (black in Fig. 6b), indicating some quenching with that time constant. Most of the quenching is with a 195 ps time constant (blue DAS in Fig. 6b), and a small fraction is unquenched (green in Fig. 6b).

**Table 1:** Estimated lifetimes (in ps) of unquenched and quenched CK, CB, and WT PB

Sample	τ1 (black)	τ2 (red)	τ3 (blue)	τ4 (green)	τ5 (magenta)
CK U	43	1680			
CK Q	25	52*	195	1360	
CB U	18	42*	112*	1190*	1680
CB Q	17	38	123	1190	
WT U	15	69	139	1480*	1680
WT Q	15	63	131	1480*	*248*

For presentation purposes, τ4 and τ5 have been interchanged for WT Q (indicated by italics)

Asterisks indicate additionally estimated lifetimes. Further explanation in text

#### Global analysis of unquenched and quenched TRES of CB PB

In Fig. 6c, d the DAS estimated from the unquenched and quenched TRES of CB is depicted. Three equilibration time constants are estimated in unquenched CB, 18, 42, and 112 ps (black, red, and blue DAS in Fig. 6c), which are attributed to intra-rod, short-rod-to-core, and whole CB PB equilibration. The green DAS represents a small fraction of unconnected rods that decays in 1.2 ns. Its shape and lifetime were linked between the unquenched and quenched data sets. Most of the quenching is with a 112 ps time constant (blue DAS in Fig. 6d). The red DAS (38 ps) is again a mixture of short-rod-to-core equilibration and quenching.

### Global analysis of unquenched and quenched TRES of WT PB

In Fig. 6e, f, the DAS estimated from the unquenched and quenched TRES of WT PB is depicted. Again, the green DASs represent a small fraction of unconnected rods that decay in 1.5 ns. As in CB, its shape and lifetime were linked between the unquenched and quenched data sets. Three equilibration time constants are estimated in unquenched WT PB, 15, 69, and 139 ps (black, red, and blue DAS in Fig. 6e), which are attributed to intra-rod, rod-to-core, and whole WT PB equilibration. Note that because of the longer rods the rod to core equilibration time constant increases from 42 (in CB PB) to 69 ps. The blue DAS in Fig. 6e is strongly non-conservative, probably due to some annihilation. In addition to this annihilation, the blue DAS in Fig. 6f shows quenching dominantly with 131 ps, with a smaller fraction decaying with 248 ps (magenta DAS in Fig. 6f). Compared to the analysis in Figure 4 of (Tian et al. 2012) several additional lifetimes have been estimated, and because of the linking of the SAS and lifetimes of the free rods their contributions have been resolved. These additional lifetimes are marked with an asterisk in Table 1.

Table 2 summarizes the main time constants estimated from TRS at different powers, and from TRES of CK, CB, and WT PB.

**Table 2:** Summary of estimated time constants of PB equilibration

Equilibration	Sample	Exc (nm)	Figure	Pigments	Time constant (ps)
Intrahexamer	WT	622	5	PC640, PC650	5
Intradisk	CB	671	3	APC660, APC680	1.6–2.6
Intracylinder	CB	671	3	APC660, APC660	13–19
Rod to core	WT	622; 590	5, S5; 6E	PC650, APC660	48,59; 69
Short rod to core	CB	590	6C	PC650, APC660	42
Intercylinder, whole PB	All	All	2, 3, 5, S2–S5, 6	All	75–200

### Target analysis of the phycobilisome

**Fig. 7:**
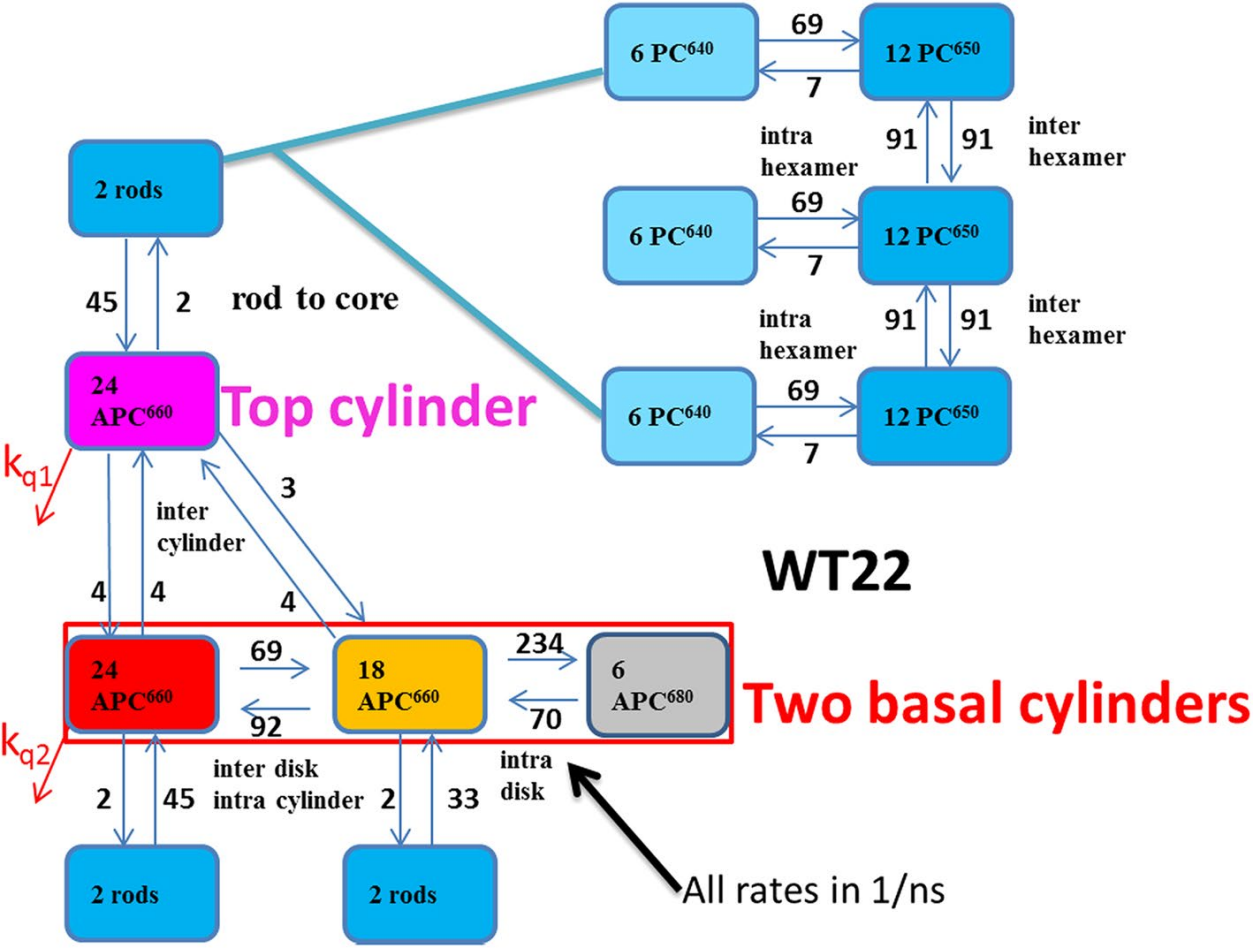
Functional compartmental model of the WT PB, with a zoom out of a rod consisting of three hexamers in the upper right. The common *k*_*fl*_ rate constant for excited states of 0.6/ns has been omitted for clarity. The quenching rates *k*_*q1*_ and *k*_*q2*_ and the equilibria are discussed in the text

The target analysis of the phycobilisome is based upon the structural model of (Arteni et al. 2009), explained in the introduction. The aim is to describe the TRS presented above as well as the TRES of (Tian et al. 2012) reanalyzed above. The kinetic scheme contains six different types of functional compartments. Because of the PB architecture that possesses C2 rotational symmetry, the core can be described by three APC660 and one APC680 compartment (black). Three APC660 compartments are needed: the top cylinder (magenta), the APC660 pigments in the D and EF disks (orange) that are in closest contact to the APC680 pigments, and the remaining APC660 pigments of the basal cylinders (red). In WT PB a rod comprises three hexamers. In turn, a hexamer comprises two types of functional compartments: PC640 (cyan) and PC650 (blue). It is assumed that two rods are connected to the top cylinder, whereas four rods radiate from the two basal cylinders. This kinetic scheme which contains 4 + 3 × 6 = 22 compartments, is termed WT22, and it is shown in Fig. 7.

The microscopic rate constants connecting the compartments must obey detailed balance. In particular, the rate constants towards the orange APC660 compartment which contains 18 pigments, are 18/24 times as large as the analogous rate constants towards the red APC660 compartment which contains 24 pigments. The inputs to the compartments must be consistent with the stoichiometry and absorption properties of the pigments, *vide infra*. The spectral properties of the different compartments are described by SADS or SAS to be estimated from the data. With time-resolved emission data, the SAS must be non-negative. In addition, penalties are used to attain equality of the areas of the SAS of pigments of equal oscillator strength. Simultaneous target analysis of data measured in conditions without and with OCP-related energy quenching provides additional information on the PB dynamics. This energy quenching is described by additional decays from the red and magenta compartments (*vide infra*). The parameters of this target model are estimated from the time-resolved emission and difference absorption spectra. Above we have globally analyzed 19 TRS data sets (3 CK, 13 CB, and 3 WT) and 8 TRES data sets (2 CK, 2 CB, and 4 WT). Consistently fitting all 27 datasets is a herculean task. This task was split in subtasks: related experiments were analyzed simultaneously, e.g., 3 TRS of CK PB (Figure S15), 6 TRS of CK PB (Figure S9), 3 TRS of WT PB (Figure S14), U and Q TRS of CB PB (Figure S11, Figure S13), U and Q TRES of CK PB (Figure S 20), CB PB (Figure S19) or WT PB (Figure S16, Figure S17). The kinetic scheme used in each of these simultaneous target analyses was consistent with that of Fig. 7. Iteratively the rate constants depicted in Fig. 7 were estimated for the WT PB. Based upon hundreds of fits, the estimated relative precision of each rate constant is 20%.

**Fig. 8:**
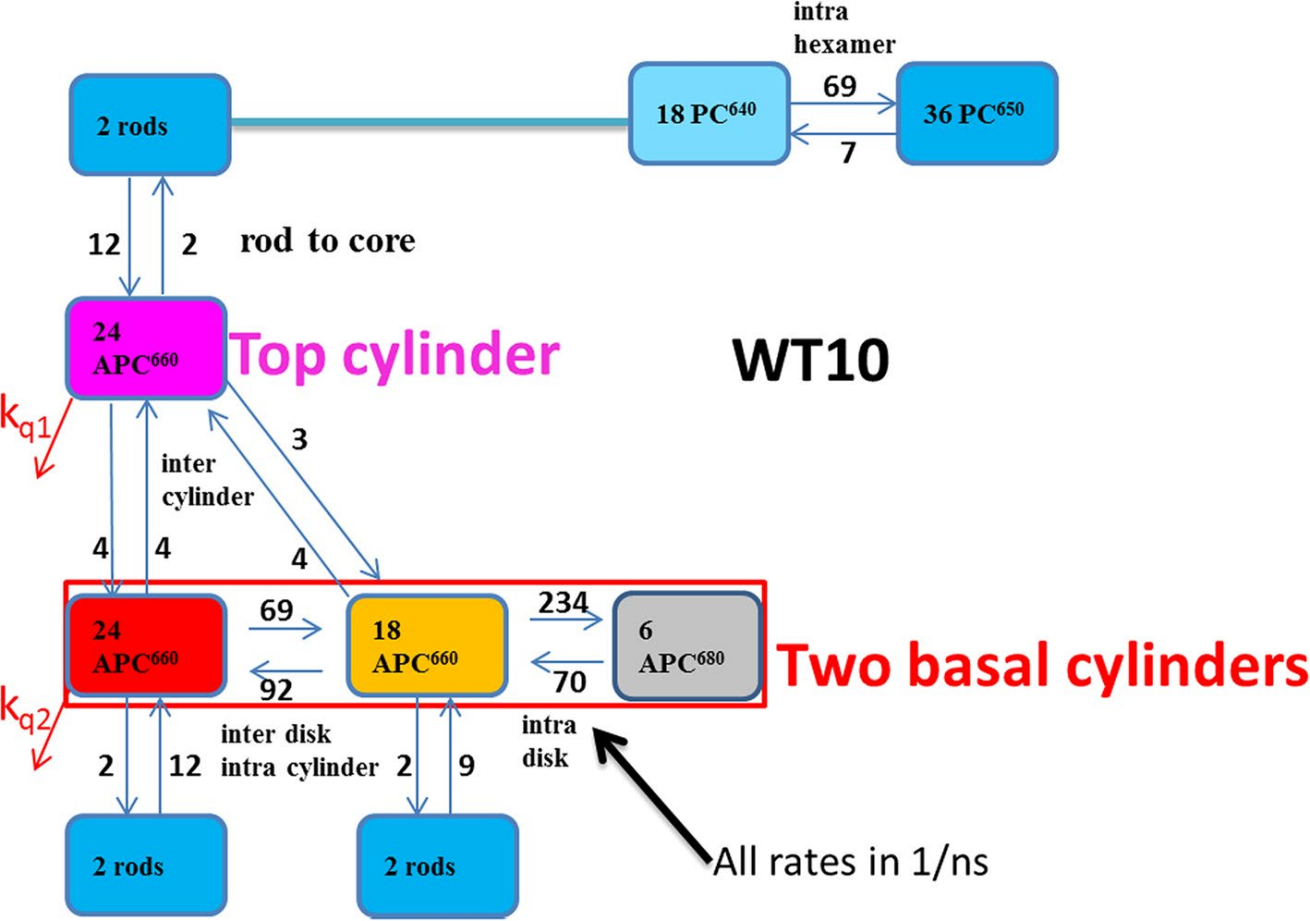
Alternative functional compartmental model of the WT PB, with a zoom out of a rod consisting of three lumped hexamers in the upper right. The common *k*_*fl*_ rate constant for excited states of 0.6/ns has been omitted for clarity

Alternatively, assuming very fast equilibration in the rods, each can be described with two compartments (PC640 and PC650). This kinetic scheme which contains only ten compartments is therefore termed WT10, and it is shown in Fig. 8. The kinetic scheme for CB, which contains only short rods, cf. Figure S6, also has ten compartments. From the difference in the rod to core equilibration times between WT and CB (cf. Table 2), an interhexamer equilibration rate can be inferred. This rate was estimated to be 91/ns.

**Fig. 9:**
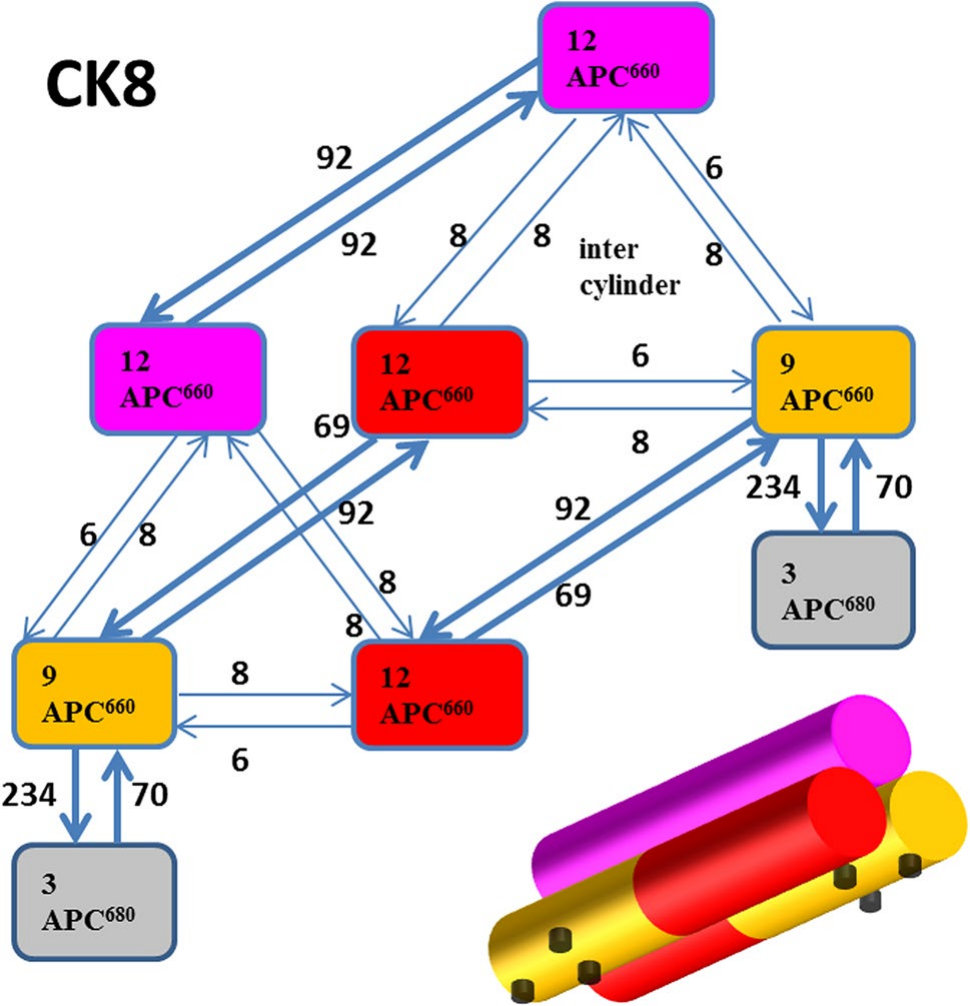
Functional compartmental model without taking into account the symmetry of CK PB, viewed from below the membrane. In the bottom right, the three *cylinders* are sketched, cf. Fig. 1. Each *half cylinder* contains two disks, thus 12 pigments. All rates are in 1/ns. *Thick arrows* indicate the fast EET rates inside a disk (between APC660 *orange* and APC680) and within a cylinder (between the APC660 *magenta* compartments, and between APC660 *orange* and APC660 *red*). Thin *arrows* indicate the intercylinder slow EET rates. The common rate constant for excited states of 0.6/ns has been omitted for clarity, and the quenching rates have also been omitted. Further explanation in text

The most difficult rate to estimate is that connecting the top and basal cylinders. It is responsible for the slowest equilibration time constant in the whole PB. Estimates for this rate varied from 2 to 10/ns, and ultimately it was fixed to 4/ns, which corresponds to a time constant of 111 ps in CK. This is still 15 times faster than the natural lifetime of 1650 ps of the pigments, thus the top cylinder still makes a significant contribution to the light harvesting. When the C2 rotational symmetry is not taken into account, the number of compartments doubles. This becomes difficult to visualize for WT PB, but is still doable for CK PB, which because of the doubling then contains eight compartments. Figure 9 shows this CK8 functional compartmental scheme for an unquenched CK PB. Note that, along each cylinder the equilibration is fast, but the intercylinder rates are now 6 and 8/ns (depending upon the number of APC660 pigments in the compartments). The longest equilibration time constant in CK8 is 118 ps, which is very close to the 111 ps in CK4 (Fig. 12a). Thus, the rate constants of 3 and 4/ns in a kinetic scheme that takes into account the symmetry (e.g., Fig. 7) have to be interpreted as effective rate constants. The “true” microscopic rate constants are twice as large, since now also the two basal cylinders have to equilibrate. We have verified that the computationally faster fit using the CK4 kinetic scheme that takes into account the symmetry, cf Fig. 12a, is fully equivalent to the fit using the CK8 kinetic scheme of Fig. 9.

The ratio of the rate constants between the gray and orange compartments was calculated as follows: the energy difference between the assumed maxima of 660 and 681 nm is 470/cm or 58 meV. The entropy difference between compartments with 18 and 6 pigments is ${k_B}T\ln ({N_1}/{N_2})={k_B}T\ln (3)$ which is 28 meV at room temperature. Thus, the free energy of the gray compartment is assumed to be 58 − 28 = 30 meV lower than that of the orange compartment, which corresponds to a ratio of the backward to forward rate constants of 0.3. Thus, the number of rate parameters to be estimated in unquenched systems is nine with WT22, eight with WT10 or CB10, five with CB7, and three with CK4 (*vide infra*, Fig. 12a). The equilibration rates within the basal cylinders have been estimated from the measurements with the highest time resolution and SNR, which are the transient absorption measurements at different powers (Fig. 3, Figure S9). Since these measurements showed different amounts of annihilation, it was necessary to include that in the target model.

### Target analysis of annihilation

In order to model the strong annihilation present in the TRS measurements with core excitation as demonstrated in Fig. 3, we assume that next to the annihilation free PB of Figure S7 (where each compartment decays only with its natural decay rate $k_{f}^{{}}$), there are three more occurrences of the PB compartmental scheme. In each of these occurrences, the annihilation is modeled as an additional decay rate from the four core compartments (Figure S8). These three annihilation time constants were estimated to be 2.9, 25.5, and 147 ps, and roughly represent, respectively, intradisk, interdisk/intracylinder, and intercylinder annihilation. With increasing laser power, the fraction of annihilation free PB decreases, and in particular the fraction of 25.5 ps (interdisk/intracylinder) annihilation increases, cf. Table S5. The fit quality of this target model is excellent, cf. Figure S9. The estimated concentrations and SADS depicted in Figure S10 are realistic. Note that, the longest annihilation time constant of 147 ps is close to the longest equilibration time constant in the core (*vide supra*, 118 ps in CK8) and the longest equilibration time constant of 127 ps in the annihilation free CB PB (CB7, Figure S7).

### Target analysis of energy quenching

In our previous work (Tian et al. 2011, 2012), we demonstrated that the APC660 pigments are being quenched in the presence of OCP^r^. Now that we have a core model with three APC660 compartments, we can estimate the quenching rate of each of these compartments. It turns out that ${k_q}$ of the orange APC660 compartment (that is in fast equilibrium with APC680) is estimated to be practically zero. For simplicity, we first assume that ${k_{q1}}$ and ${k_{q2}}$ are the same, and will here be called ${k_q}$. In WT PB two quenching rates are present, with the smallest rate being similar to the rate estimated with CK PB or CB PB 5 nJ (cf. Table 3). This suggests that the PB contains more than one quenching site, and that the quenching rate is proportional to the occupation of these quenching sites. Even a small ${k_q}$ rate of ≈19/ns is sufficient to efficiently quench excess excitations. This is obvious from the comparison of the DAS in Fig. 6.

**Table 3:** Estimated fraction unquenched, annihilation free, and quenching rate in 1/ns for the different TRES and TRS experiments

Measurement	TRES	TRES	TRES	TRS	TRS	TRS
Sample	CK	CB	WT	CB 2 nJ	CB 3 nJ	CB 5 nJ
% Unquenched	17	1	2	71	39	25
${k_q}$(1/ns)	19	54	14 (29%) 100 (71%)	26	36	16
% Annihilation free	100	100	85	61	49	29

The fit quality of this target model of energy quenching is good, cf. Figure S11 for the CB 5 nJ TRS data. The estimated concentrations and SADS depicted in Figure S12 for the CB TRS data are realistic. The SADS estimated with the three different powers are very similar.

**Fig. 10:**
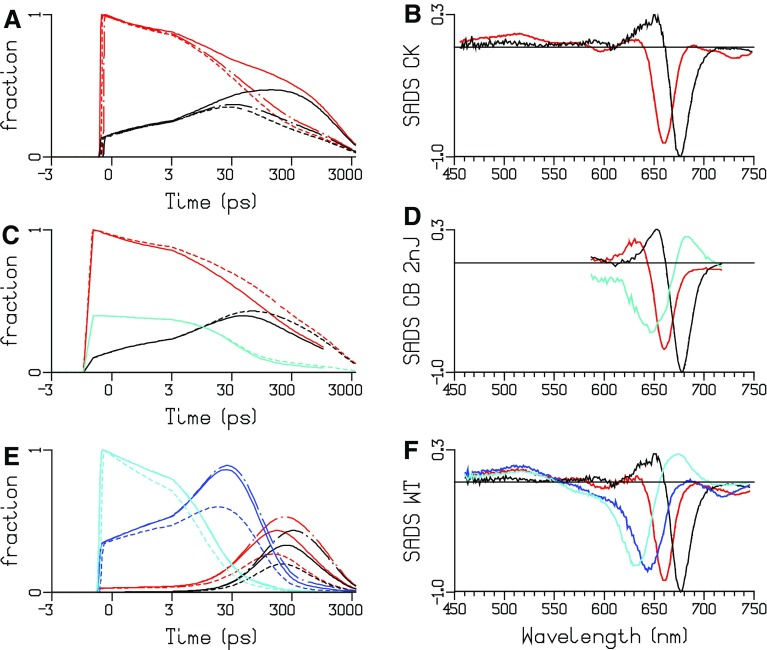
Total concentrations and SADS estimated from TRS of CK PB (**a, b**, 656 nm exc), CB PB (**c, d**, 650 nm exc, 2 nJ, kinetic scheme CB7, Figure S7) and WT PB (**e, f**, 622 nm exc, kinetic scheme WT10, Fig. 8). Key: PC640 (*cyan*), PC650 (*blue*), short rod (*turquoise*), APC660 (*red*), and APC680 (*black*). **a** 2 nJ (*solid*), 8 nJ (*dot* dashed), 12 nJ (*dashed*). **b** Unquenched (*dashed*), quenched (*solid*). **c** 2 nJ (*dot dashed*), 5 nJ (*solid*), 10 nJ (*dashed*). Note that, the time axis is linear until 3 ps and logarithmic thereafter

The estimated SADS for all TRS data depicted in Fig. 10 are consistent with the properties of the four types of pigments. With WT PB, the maxima of the bleach plus SE are at 629, 643, 660, and 676 nm for, respectively, PC640 (cyan), PC650 (blue), APC660 (red), and APC680 (black). The SADS of APC660 (red) and APC680 (black) are consistent between CK, CB, and WT PB. The good quality of the fit of the TRS data is demonstrated in Figures S11, S13, S14, and S15. The estimated scaling parameters for the data sets were consistent with the powers that were used in the experiments (Figures S9, S14, S15). In panels a, c, and e of Fig. 10, the total concentration is plotted. For each species, the total concentration is the sum of all excited state populations in the compartments with the spectrum of that species. Thus, the populations of the three APC660 compartments are summed, etc. Different line types indicate the experiments at different powers in panels a and e, and of unquenched and quenched experiments in panel c. The increase of the annihilation with increasing power is clearly visible in Fig. 10a, e. The estimated microscopic rate constants of Figs. 7 and 8 range from three till 234/ns. These rates can be attributed to Förster EET. The estimate rates within and between hexamers (69 and 91/ns) are consistent with calculations of Förster EET rates in rods (Xie et al. 2002). Recently, sub-ps EET from PC to APC has been claimed (Nganou et al. 2016). This most probably is a misinterpretation of scarce data. The kinetic schemes of Figs. 7 and 8 are consistent with earlier studies reviewed in Holzwarth (1991) and van Grondelle et al. (1994).

**Fig. 11:**
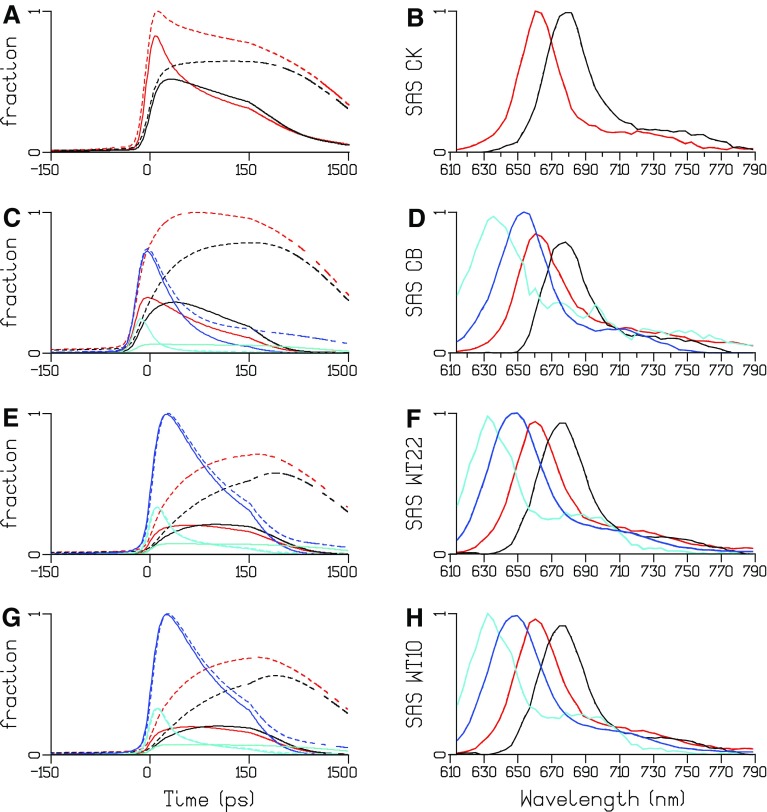
Total concentrations and SAS estimated from TRES after 590 nm exc of CK PB (**a, b**), CB PB (**c, d**, kinetic scheme CB10, Figure S6) and WT PB (**e, f**, kinetic scheme WT22, Fig. 7, and GH, kinetic scheme WT10, Fig. 8). Key: PC640 (*cyan*), PC650 (*blue*), APC660 (*red*) and APC680 (*black*), unquenched (*dashed*), quenched (*solid*). *Turquoise* indicates the fraction of free rods, which possess the PC650 SAS. Note that the time axis is linear until 150 ps and logarithmic thereafter

The estimated SAS for all TRES data depicted in Fig. 11 are consistent with the properties of the four types of pigments. With WT PB, the maxima of the emission are at 635, 649, 660 and 682 nm for, respectively, PC640 (cyan), PC650 (blue), APC660 (red), and APC680 (black). The SAS of APC660 (red) and APC680 (black) are consistent between CK, CB, and WT PB. The fit quality of the target analysis of the TRES data is good, cf. Figures S16, S19, and S20. With WT, the quality of the fit is not significantly different for the WT22 and WT10 kinetic schemes. Only very subtle differences are visible when overlaying, cf. Figure S18. Thus, depending upon the purpose, both kinetic schemes WT22 and WT10 can be accepted.

### Relative absorption of the pigments

Next to the kinetic scheme and its parameters also the relative absorptions of the different pigments with the different excitation wavelengths have to be estimated. With TRES, the time zero spectrum is the sum of the SAS of all the excited pigments. Thus, the shape of the SAS of the fastest decaying species, PC640, is the most sensitive to the relative absorption parameters. The parameters of Table 4 that led to the acceptable SADS of Fig. 10 and SAS of Fig. 11 were thus determined iteratively. Based upon this relative absorption, the percentage excitation of each pigment type can be calculated. Both numbers are collated in Table 4.

The amplitude matrix of the WT10 target analysis (Fig. 8) of the unquenched TRES data is shown in Table 5. The fastest equilibration is between the orange APC660 and APC680 species, 2.5 ps (purple shading). Then with a 13 ps time constant, the PC640 and PC650 pigments equilibrate, as well as the core basal cylinders (yellow and orange shading). The first rod to core equilibration time constant is 68 ps (blue shading). The subsequent rod to core equilibration time constants are 89 and 115 ps (green shading). This agrees with the results from (Suter and Holzwarth 1987). Intercylinder equilibration is visible as the decay of the magenta APC660 with time constants of 115 and 148 ps. The latter time constant is consistent with the slowest annihilation time constant of 147 ps (Table S5).

**Table 4:** Percentage excitation of pigment type and between parentheses the relative absorption of a single pigment with the different excitation wavelengths and experiments

Excitation (nm)	Experiment	PC640	PC650	Free rod	APC660	APC680
590	CK TRES	–	–		92% (1)	8% (1)
590	CB TRES	32% (2)	32% (1)	3% (1)	30% (1)	3% (1)
590	WT TRES	41% (2)	41% (1)	4% (1)	13% (1)	1% (1)
622	WT TRS	73.5% (6)	24.5% (1)		2% (0.08)	–
650	CB TRS	–	27% (0.5*)		67% (1)	6% (1)
656	CK TRS	–	–		89% (1)	11% (1.4*)
671	CB TRS	–	3–10% (0.04*)		72 − 69% (1)	25 − 21% (4)

*Indicates estimated

**Table 5:**
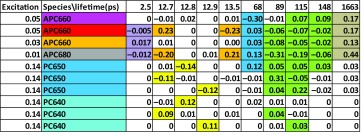
Amplitude matrix of the WT10 unquenched analysis with 590 nm excitation

Color code of the species and estimated microscopic rates corresponds are given in Fig. 8. Color code of the largest amplitudes indicates equilibration between compartments, except for the last column where it shows that 44% of the excitations ends up in APC680, and 47% in APC660. Further explanation in the text

### Energy quenching in the CK PB emission revisited

**Fig. 12:**
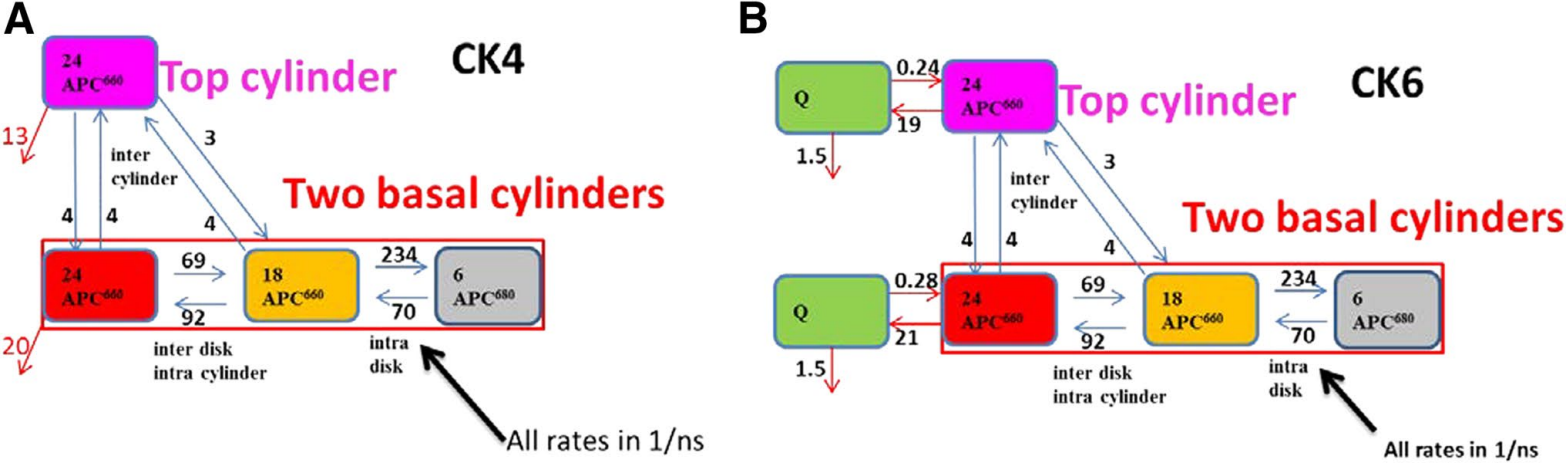
Kinetic schemes CK4 (**a**) and CK6 (**b**) for energy quenching in CK PB. The common rate constant for the APC660 and APC680 excited states of 0.6/ns has been omitted for clarity

**Fig. 13:**
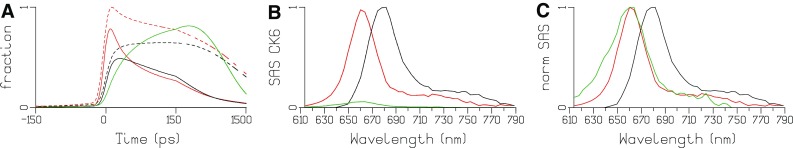
Total concentrations (**a**), SAS (**b**) and normalized SAS (**c**) estimated from TRES after 590 nm exc of CK PB using the kinetic scheme CK6, Fig. 10b. Key: Q (*green*), APC660 (*red*) and APC680 (*black*), unquenched (*dashed*), quenched (*solid*). Note that the time axis is linear until 150 ps and logarithmic thereafter

The TRES data of CK PB in the unquenched and quenched state provide the most direct evidence of energy quenching. In these data, there is no annihilation present (like in the CB PB TRS data, or the WT PB TRES data) and the absence of the rods allows a closer look at the more blue emission of the APC660. The SVD analysis of the residual matrix that results from the target analysis using the CK4 kinetic scheme (Fig. 12a) reveals that between 610 and 670 nm some additional long lived emission is present in the Q data (Figure S21A, B), which can also be observed directly as a lack of fit in Figure S20. First, we tested whether allowing ${k_{q1}}$ and ${k_{q2}}$ to differ improved the fit. Indeed this was the case, the rms error of the fit decreased slightly, from 1.826 to 1.820. The estimated ${k_{q1}}$ and ${k_{q2}}$ were, respectively, 13 and 20/ns. This suggests that the strongest quenching is present with the red APC660 compartment in the basal cylinders. Next an additional species called Q was added, resulting in the CK6 kinetic scheme (Fig. 12b). The rms error of the fit decreased significantly from 1.820 to 1.690, and the SVD analysis of the residual matrix using the CK6 kinetic scheme is satisfactory, cf. Figure S21E, F versus Figure S21A, B. This suggests that quenching corresponds to EET to a low lying state Q (with a free energy 112 meV below that of the red or magenta APC 660 compartment) with a rate of ≈20/ns. The area under the SAS of this new species Q is small, with oscillator strength 15 times smaller than APC660, cf. Fig. 13b, and with relatively more blue emission than APC660, cf. Fig. 13c. We also performed a target analysis of CB PB and of WT PB with a model that contained additional Q compartments, and describe these results in the Supporting Information. In both CB PB and WT PB, the rms error of the fit improved (cf. Figures S22 and S25), and a Q SAS with relatively more blue emission than APC660 was estimated, cf. the green and red SAS in Figures S24C and S27C. The nature of this new species Q, which possibly is related to the blue shifted spectrum observed in single APC trimers (Wang and Moerner 2015), remains to be further investigated.

### Simulation of equilibration and annihilation inside the core

**Fig. 14:**
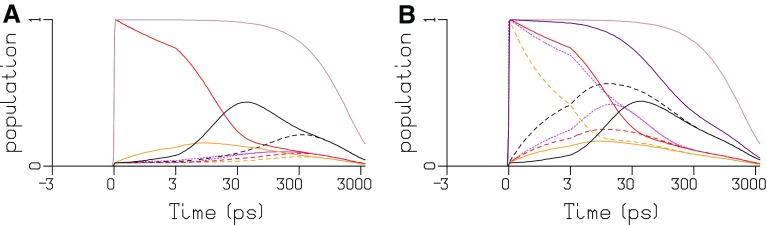
Excited state populations of all eight compartments of CK8 depicted in Fig. 9a after exciting the red APC660 compartment of the left basal cylinder, and b after additionally exciting the orange APC660 compartment of the right basal cylinder, and one *magenta* APC660 compartment of the top cylinder. Key: left basal cylinder (*solid*), right basal cylinder (*dashed*), top cylinder (*dotted*), APC660 (*red, orange* and *magenta*), and APC680 (*black*). The total fraction of excited states without (**a**) and with (**b**) annihilation is in *brown* and *maroon*, respectively. Note that, the time axis is linear until 3 ps and logarithmic thereafter

Now that we have constructed the PB functional compartmental model, we can simulate edge to edge equilibration inside the core by selectively exciting one compartment of the CK8 model depicted in Fig. 9. In Fig. 14a, the excited state populations of all eight compartments are depicted after exciting only the red APC660 compartment of the left basal cylinder. The equilibration in this cylinder (solid lines) with a time constant of 12 ps is visible as the decay of the red and rise of the orange APC660 and APC680 (black) populations. Edge-to-edge equilibration is clearly visible as the slow rise of the APC680 population in the right basal cylinder (black dashed line) with a time constant of 118 ps. It is this 118 ps time constant that determines the complete equilibration of the core, and that is responsible for the slowest annihilation time constant observed in the TRS data. To demonstrate the annihilation in the core (Fig. 2) with the CK8 model, we simulated equal excitation of three different APC660 compartments in the three cylinders in Fig. 14b. In the right basal cylinder (dashed lines), the excited APC660 (orange) equilibrates with a lifetime of 2.5 ps with the APC680 (black). The intracylinder equilibration lifetime is 12 ps, e.g., the dotted magenta lines of the APC660 in the top cylinder. The intercylinder equilibration lifetimes are 59 and 118 ps. The annihilation takes place with approximately these lifetimes, cf. the brown and maroon curves that represents the total fraction of excited states without (Fig. 14a) and with (Fig. 14b) annihilation. The fraction annihilation free core in Fig. 14a (which represents power 2 nJ) was 100%, and in Fig. 14b (corresponding to power 12 nJ) it was only 38%.

In conclusion, we have constructed a functional compartmental model of the *Synechocystis* PB and estimated the time constants of intradisk, interdisk/intracylinder and intercylinder equilibration. The common kinetic schemes of Fig. 7 (and the simpler schemes derived from this, Figs. 8, 12, and Figure S7) describe both the transient absorption and the fluorescence emission measurements. All EET processes resulting in equilibration and annihilation as well as the quenching process are described by this unified compartmental model which consists of only four compartments that functionally describe the phycobilisome core. The slowest intercylinder equilibration time constants of ≈118 ps (CK8) and 115 and 148 ps (WT10) are most probably longer than the EET time constant from the PB core to a photosystem in the thylakoid membrane. Thus, it will be difficult to estimate this inverted kinetics process.

## Electronic supplementary material

Below is the link to the electronic supplementary material.

Supplementary material 1 (PDF 7541 KB)

## Abbreviations

APC: Allophycocyanin
BG: Blue green
DADS: Decay-associated difference spectrum
DAS: Decay-associated spectrum
DOAS: Damped oscillation-associated spectrum
EADS: Evolution-associated difference spectrum
EAS: Evolution-associated spectrum
EET: Excitation energy transfer
ESA: Excited state absorption
FWHM: Full width at half maximum
FRP: Fluorescence recovery protein
IRF: Instrument response function
OCP: Orange carotenoid protein
PB: Phycobilisome
PC: Phycocyanin
rms: Root mean square
SADS: Species-associated difference spectrum
SAS: Species-associated spectrum
SE: Stimulated emission
SNR: Signal-to-noise ratio
SVD: Singular value decomposition
TRES: Time-resolved emission spectrum
TRS: Time-resolved spectrum
WT: Wild-type
