# Supplementary material for: A functional compartmental model of the Synechocystis PCC 6803 phycobilisome
Source: Photosynth Res. 2017 Jul 18;135(1):87–102. doi: 10.1007/s11120-017-0424-5 (PMC5784004; doi:10.1007/s11120-017-0424-5)
Supplement: Supplementary file 1 — Supplementary material 1 (PDF 7541 KB) [file 11120_2017_424_MOESM1_ESM.pdf]

Supplementary Material to A functional compartmental model of the *Synechocystis* PCC 6803 phycobilisome

Ivo H.M. van Stokkum[a], Michal Gwizdala[a,b], Lijin Tian[a,c], Joris J. Snellenburg[a], Rienk van Grondelle[a], Herbert van Amerongen[c], Rudi Berera[a,d]

[a] Institute for Lasers, Life and Biophotonics, Faculty of Sciences, VU University Amsterdam, De Boelelaan 1081, 1081 HV, Amsterdam, The Netherlands

[b] Department of Physics, University of Pretoria, South Africa

[c] Laboratory of Biophysics, Wageningen University, The Netherlands

[d] Department of Food Sciences, Faculty of Agriculture, Kagawa University, Miki-cho, Kagawa 761-0795, Japan

Table of Contents

Three-dimensional structure of a trimer of allophycocyanin ... 2
Time-resolved difference absorption spectroscopy: Global analysis ... 3
Unquenched phycobilisome core at 3 different powers (CK PB) ... 3
Unquenched phycobilisome core with short rods at 6 different powers (CB PB) ... 4
Quenched and unquenched phycobilisome core with short rods (CB PB) ... 5
Unquenched phycobilisome at 3 different powers (WT PB) ... 5
Kinetic schemes for CB PB target analysis ... 6
Time-resolved difference absorption spectroscopy: Target analysis results ... 7
Unquenched phycobilisome core with short rods at 6 different powers (CB PB) ... 7
Quenched and unquenched phycobilisome core with short rods (CB PB) ... 9
Unquenched phycobilisome at 3 different powers (WT PB) ... 12
Unquenched phycobilisome core at 3 different powers (CK PB) ... 13
Time-resolved Emission spectroscopy: Target analysis results ... 14
Quenched and unquenched phycobilisome (WT PB) ... 14
Quenched and unquenched phycobilisome core with short rods (CB PB) ... 17
Quenched and unquenched phycobilisome core (CK PB) ... 18
Energy quenching in the CK PB emission revisited ... 19
Energy quenching in the CB PB emission revisited ... 20
Energy quenching in the WT PB emission revisited ... 22
References ... 24

Three-dimensional structure of a trimer of allophycocyanin

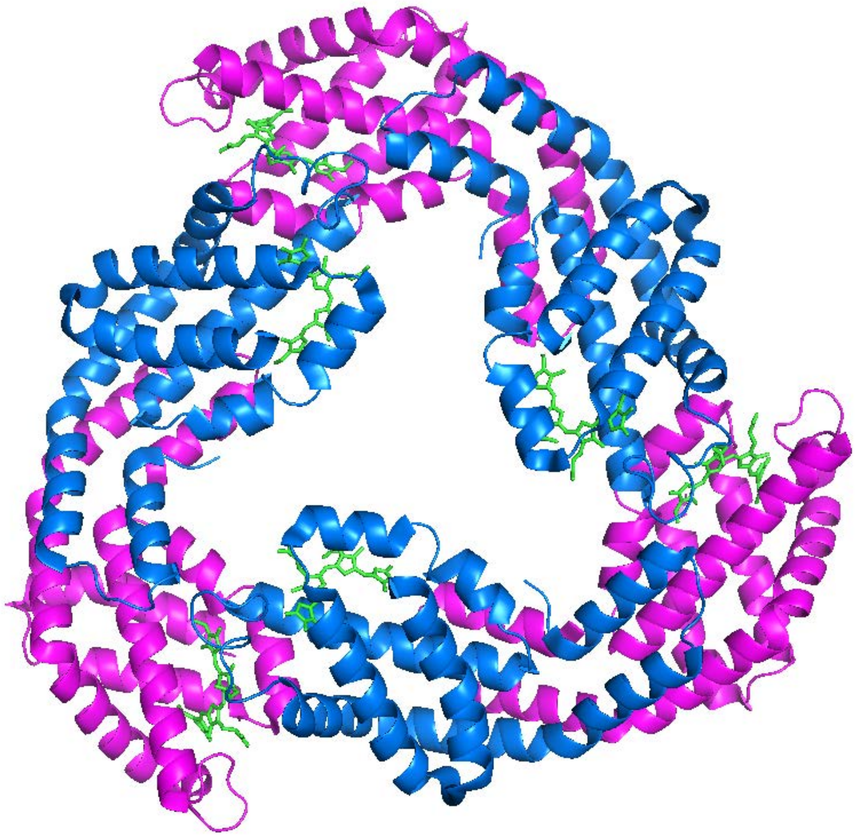

Figure S 1. Three-dimensional structure of a trimer of allophycocyanin. In the core of a PB there are three cylinders, each composed of four trimers. Each trimer is composed of three monomers and each monomer is built of α-(pink) and β-(blue) allophycocyanin subunits. Phycobilins (green) in one monomer are ≈50 Å from each other while phycobilins of two neighbouring monomers are closer (≈20 Å). The α-allophycocyanin subunits (pink) are more external than the β-allophycocyanin (blue) subunits. Picture was redrawn with PyMol from (Reuter et al. 1999) (for clarity the structure of a capping linker L_C is not shown).

Time-resolved difference absorption spectroscopy: Global analysis

Unquenched phycobilisome core at 3 different powers (CK PB)

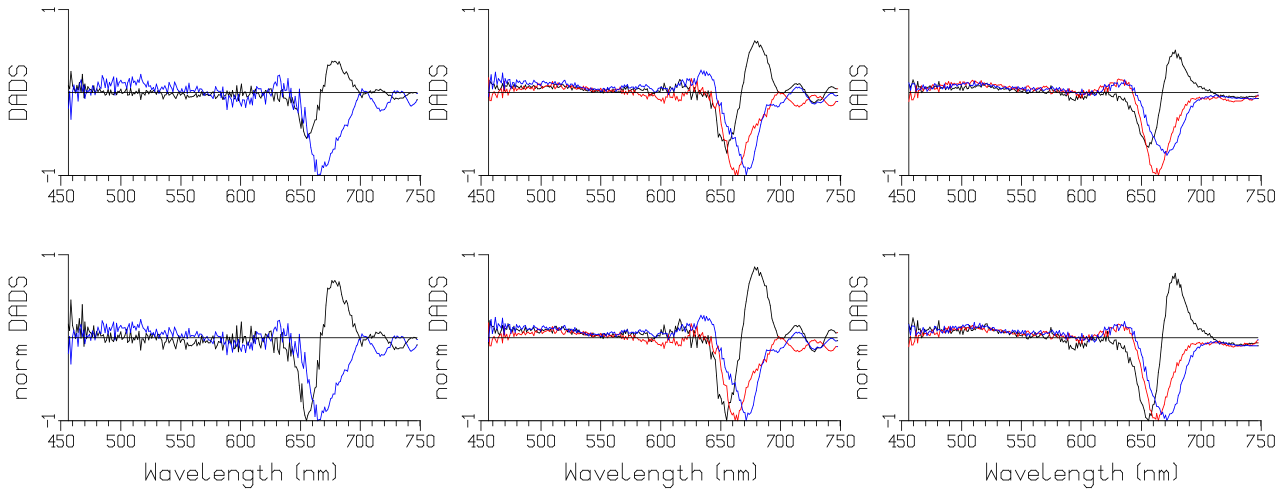

Figure S 2. Decay associated difference spectra (DADS) of CK PB after 2 (left), 8 (middle) and 12 nJ (right) excitation at 656 nm. At the bottom normalized DADS. Lifetimes collated in Table S 1. Color key of increasing lifetimes: black, red, blue. Note the annihilation (red DADS) visible as a loss of bleach plus SE peaking at 660 nm on time constants of 91 ps (8 nJ), and 75 ps (12 nJ).

Power (nJ)	τ1 (black)	τ2 (red)	τ3 (blue)
2	14	-	1700
8	9	91	1700
12	9	75	1400

Table S 1. Lifetimes (in ps) of CK PB after excitation at 656 nm with powers of 2, 8 and 12 nJ.

Unquenched phycobilisome core with short rods at 6 different powers (CB PB)

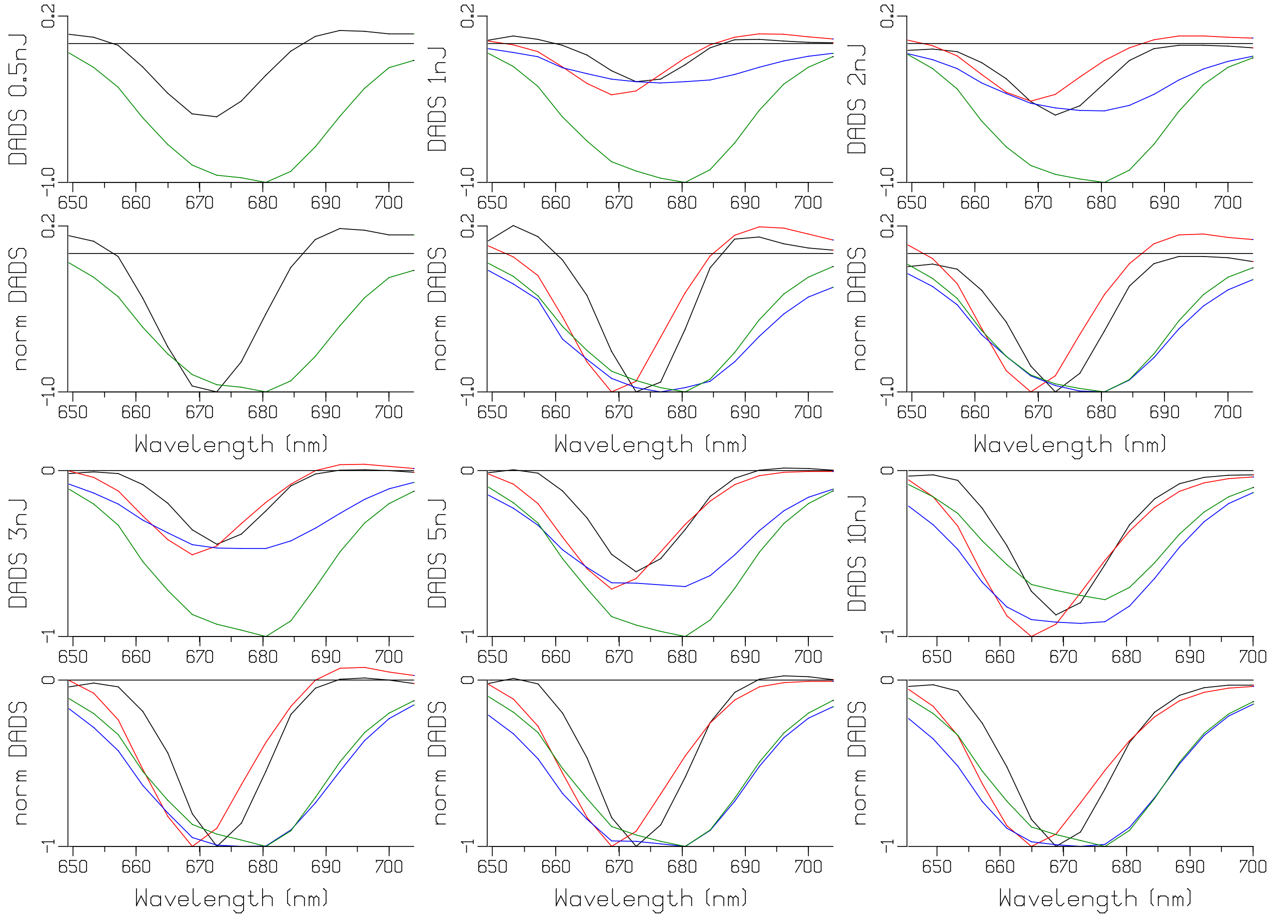

Figure S 3. DADS and normalized DADS of CB PB after excitation at 671 nm. Power of 0.5 -10 nJ indicated as ordinate label. Lifetimes collated in Table S 2. Color key of increasing lifetimes: black, red, blue, green. The final SADS (green) is similar in all six experiments. With increasing power the loss of bleach plus SE increases monotonically.

Power (nJ)	τ1 (black)	τ2 (red)	τ3 (blue)	τ4 (green)
0.5	6.7	-	-	1400
1	1.6	13	131	1300
2	2.6	17	163	1400
3	2.0	14	139	1500
5	2.2	17	138	1500
10	2.5	19	138	1500

Table S 2. Lifetimes (in ps) of CB PB after excitation at 671 nm with a power of 0.5 -10 nJ.

Quenched and unquenched phycobilisome core with short rods (CB PB)

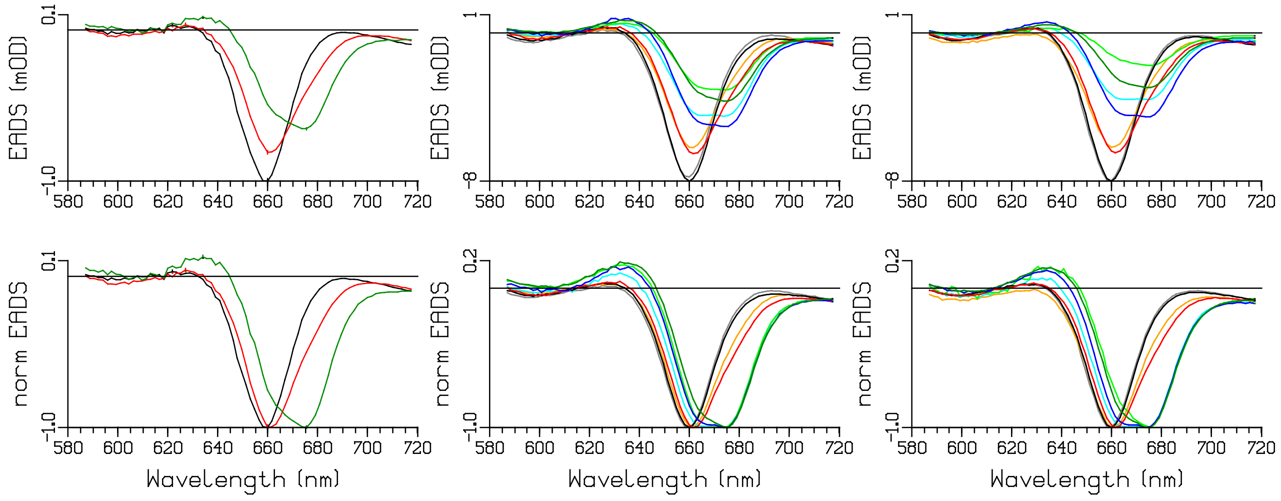

Figure S 4. Evolution associated difference spectra (EADS) and normalized EADS of CB PB after 0.5 (left), 2 (middle) and 3 nJ (right) excitation at 650 nm. Lifetimes are collated in Table S 3. Color key of increasing lifetimes: black, red, blue, dark green (unquenched) and grey, orange, cyan, light green (quenched).

Power (nJ)	τ1 (black)	τ2 (red)	τ3 (blue)	τ4 (green)
0.5	3.6	45		1500
2	4.6	34	123	1760
2 (Q)	3.5	25	65	1020
3	4.1	31	133	1670
3 (Q)	2.6	17	92	660
5	3.9	26	122	1450
5 (Q)	4.9	29	173	1430

Table S 3. Lifetimes (in ps) of CB PB after excitation at 650 nm with powers of 0.5 -5 nJ (unquenched conditions), and 2-5 nJ (Q: partly quenched conditions).

Unquenched phycobilisome at 3 different powers (WT PB)

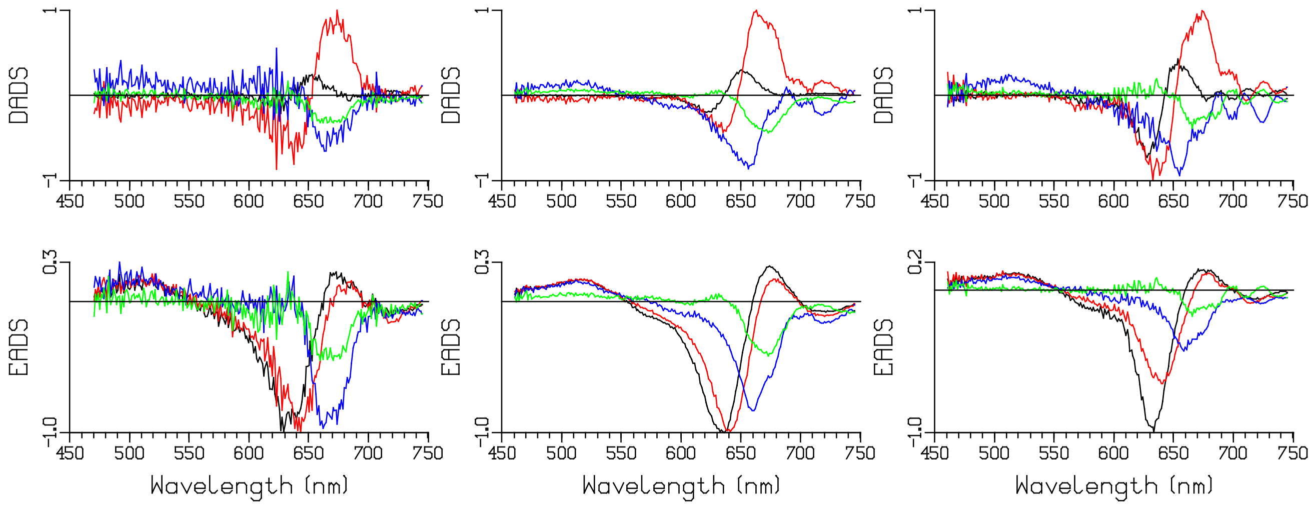

Figure S 5. DADS and EADS of WT PB after 2 (left), 5 (middle) and 10 (right) nJ excitation at 622 nm. Lifetimes are collated in Table S 4. Color key of increasing lifetimes: black, red, blue, green. Note the annihilation visible as a loss of bleach plus SE between the blue and green EADS and of the blue DADS. At 10 nJ annihilation is present at 5 and 48 ps as well.

Power (nJ)	τ1 (black)	τ2 (red)	τ3 (blue)	τ4 (green)
2	9	89	200	1700
5	5	59	132	1200
10	5	48	184	1800

Table S 4. Lifetimes (in ps) of WT PB after excitation at 622 nm with powers of 2, 5 and 10 nJ.

Kinetic schemes for CB PB target analysis

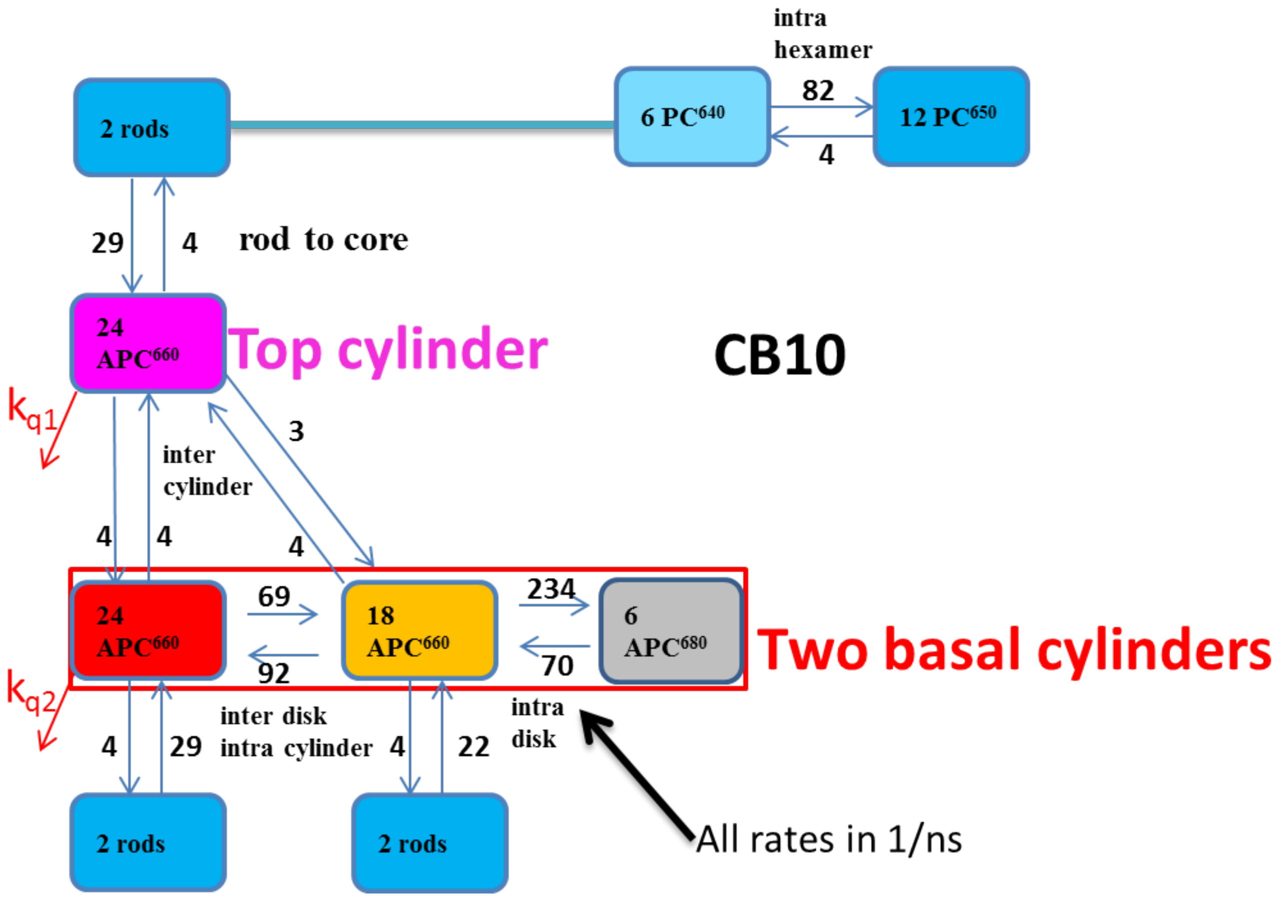

Figure S 6. Functional compartmental model of the CB PB, with a zoom out of a short rod consisting of a single hexamer in the upper right. The common k_{fl} rate constant for excited states of 0.6/ns has been omitted for clarity.

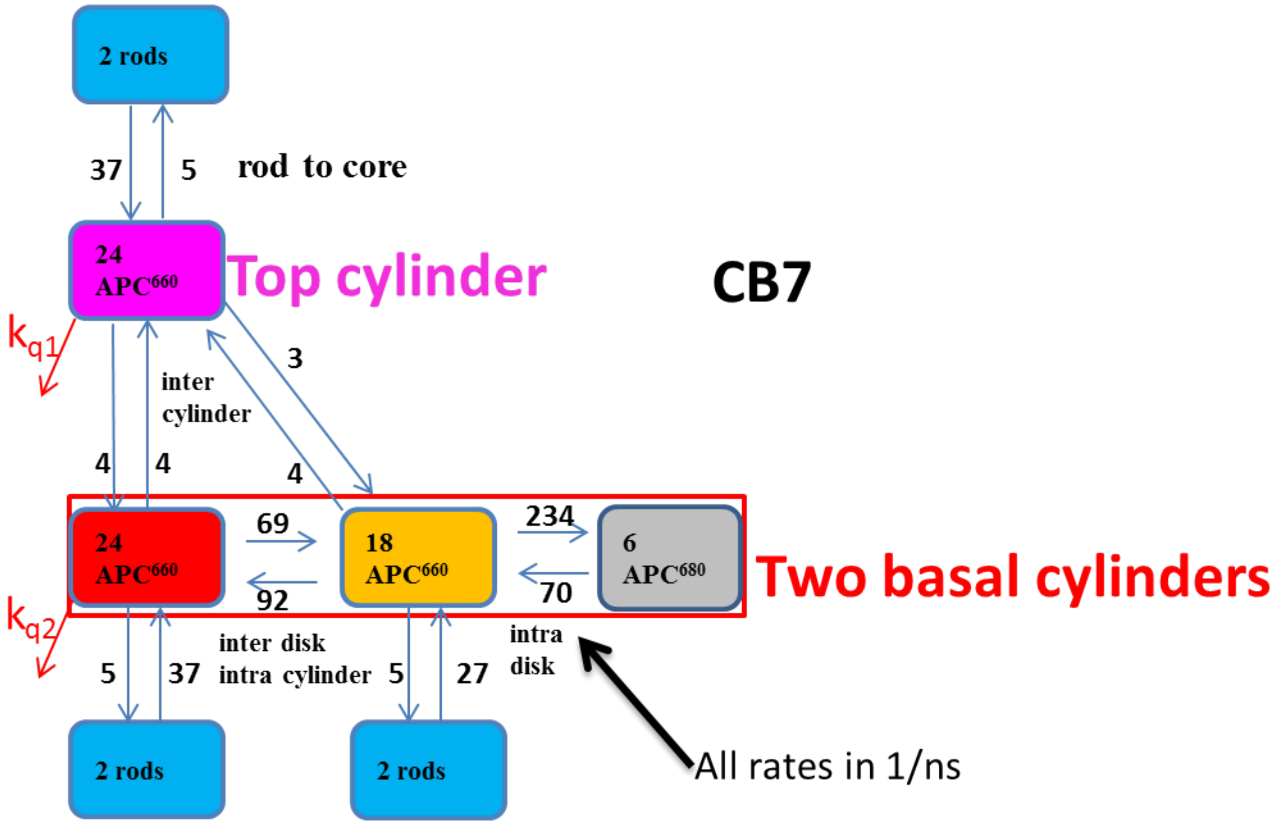

Figure S 7. Functional compartmental model of the CB PB used to fit the TRS measurements with core excitation. The common k_{fl} rate constant for excited states of 0.6/ns has been omitted for clarity.

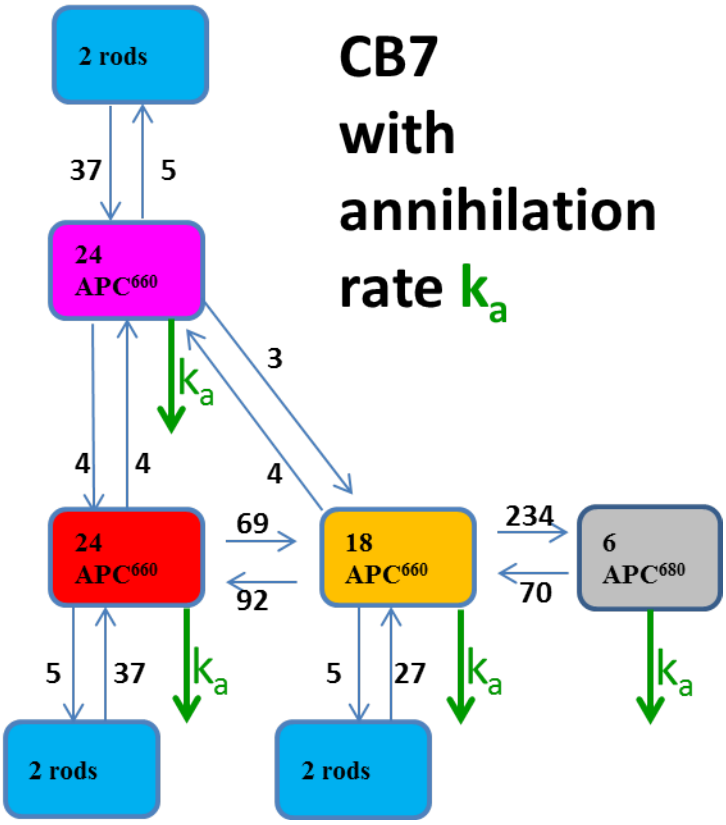

Figure S 8. Functional compartmental model of the CB PB used to fit the TRS measurements with core excitation. The common k_{fl} rate constant for excited states of 0.6/ns has been omitted for clarity. Annihilation in a complex is modelled as an additional decay rate from the four core compartments, k_a (indicated in green). A complex with $k_a = 348/ns$ (corresponding to 2.9 ps) thus experiences intradisk annihilation. A complex with $k_a = 6.8/ns$ (corresponding to 147 ps) thus experiences intercylinder annihilation.

Time-resolved difference absorption spectroscopy: Target analysis results

Unquenched phycobilisome core with short rods at 6 different powers (CB PB)

τ anni \ power	1 nJ	2 nJ	3 nJ	5 nJ	10 nJ
**2.9**	7.6%	13.9%	12.4%	13.9%	18.4%
**25.5**	1.5%	4.2%	10.7%	16.6%	24.9%
**147.3**	25.6%	28.2%	23.5%	27.3%	29.4%
annihilation free	65.3%	53.7%	53.4%	42.1%	27.3%

Table S 5. Fractions of CB PB experiencing annihilation (671 nm excitation, cf. Figure S 3) as a function of power and annihilation lifetime (in ps), cf. Figure S 8. It is assumed that 0.5 nJ corresponds to 100% annihilation free.

In the following figures we show all data (in light colors) and the target analysis fit (in dark colors) in small panels for each detection wavelength. Inspection of such a panel (possibly zoomed when viewing a pdf file) allows judgement of both the SNR and the quality of the target analysis fit.

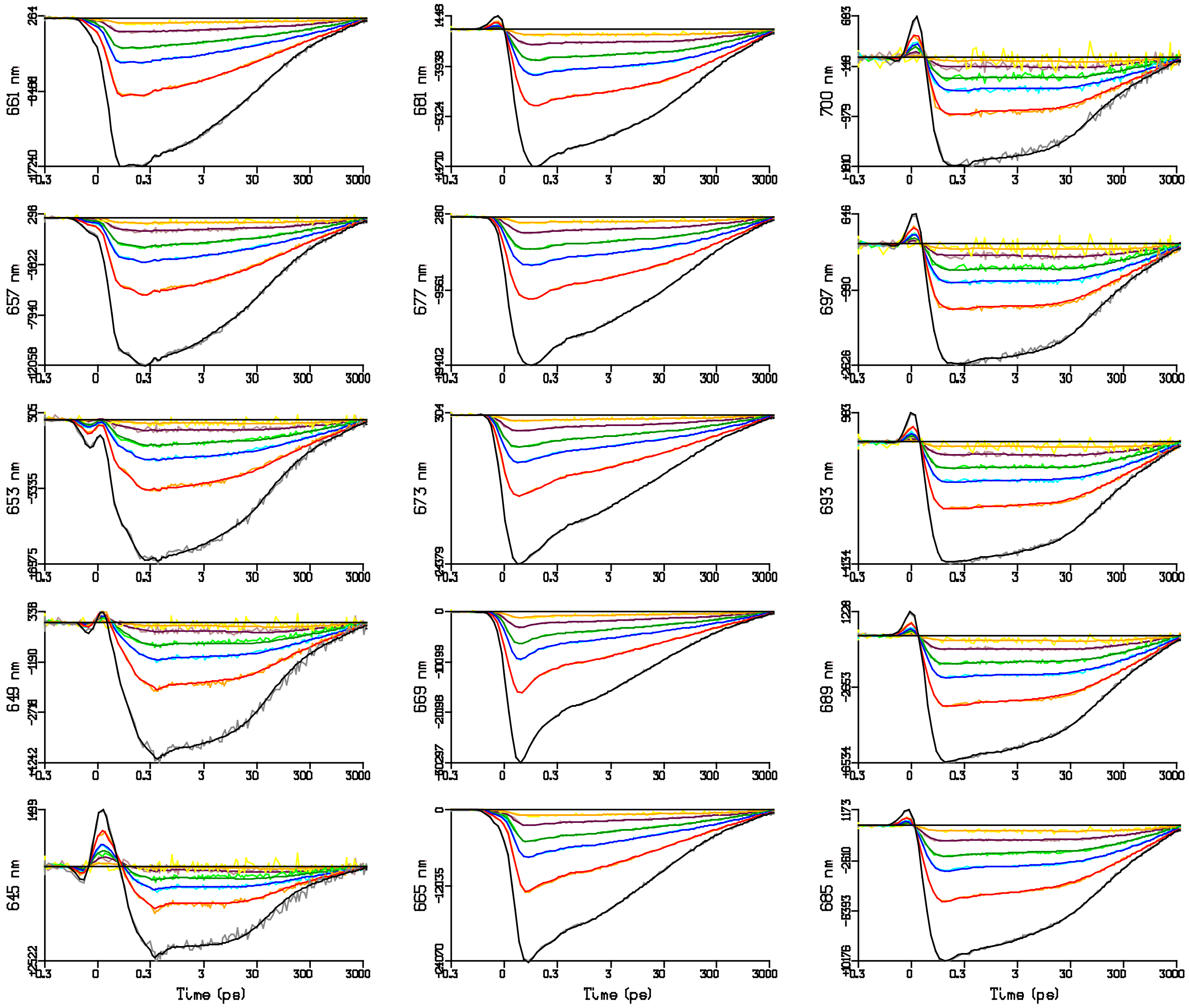

Figure S 9. Transient absorption (in units of µOD) of CB_PB at 15 wavelengths (indicated in the abscissa label) after 671 nm excitation. Key: 10 nJ (grey), 5 nJ (orange), 3 nJ (cyan), 2 nJ (light green), 1 nJ (brown) and 0.5 nJ (yellow). Black, red blue, dark green, maroon and orange lines indicate the simultaneous target analysis fit. Note that the time axis is linear until 0.3 ps and logarithmic thereafter. Note also that each panel is scaled to its maximum. Overall rms error of the fit was 67 µOD.

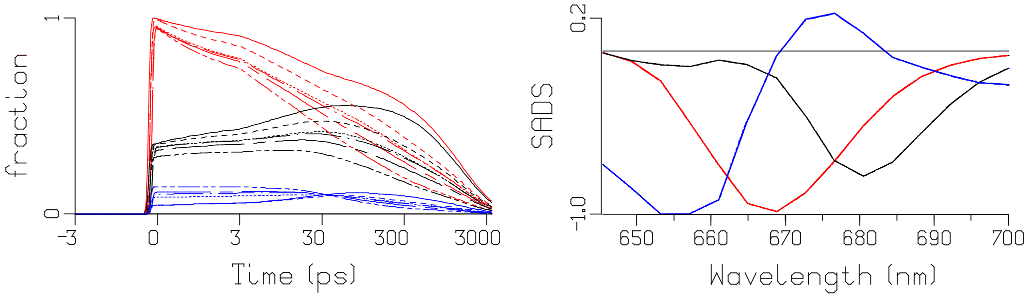

Figure S 10. Total concentrations of APC660 (red), APC680 (black) and rod (blue) and estimated SADS. Key: 10 nJ (double dot dashed), 5 nJ (chain dashed), 3 nJ (dot dashed), 2 nJ (dotted), 1 nJ (dashed) and 0.5 nJ (solid). With increasing power the percentage of rod excitation increases relative to core excitation, most probably due to saturation effects. Note that the time axis is linear until 3 ps and logarithmic thereafter.

Quenched and unquenched phycobilisome core with short rods (CB PB)

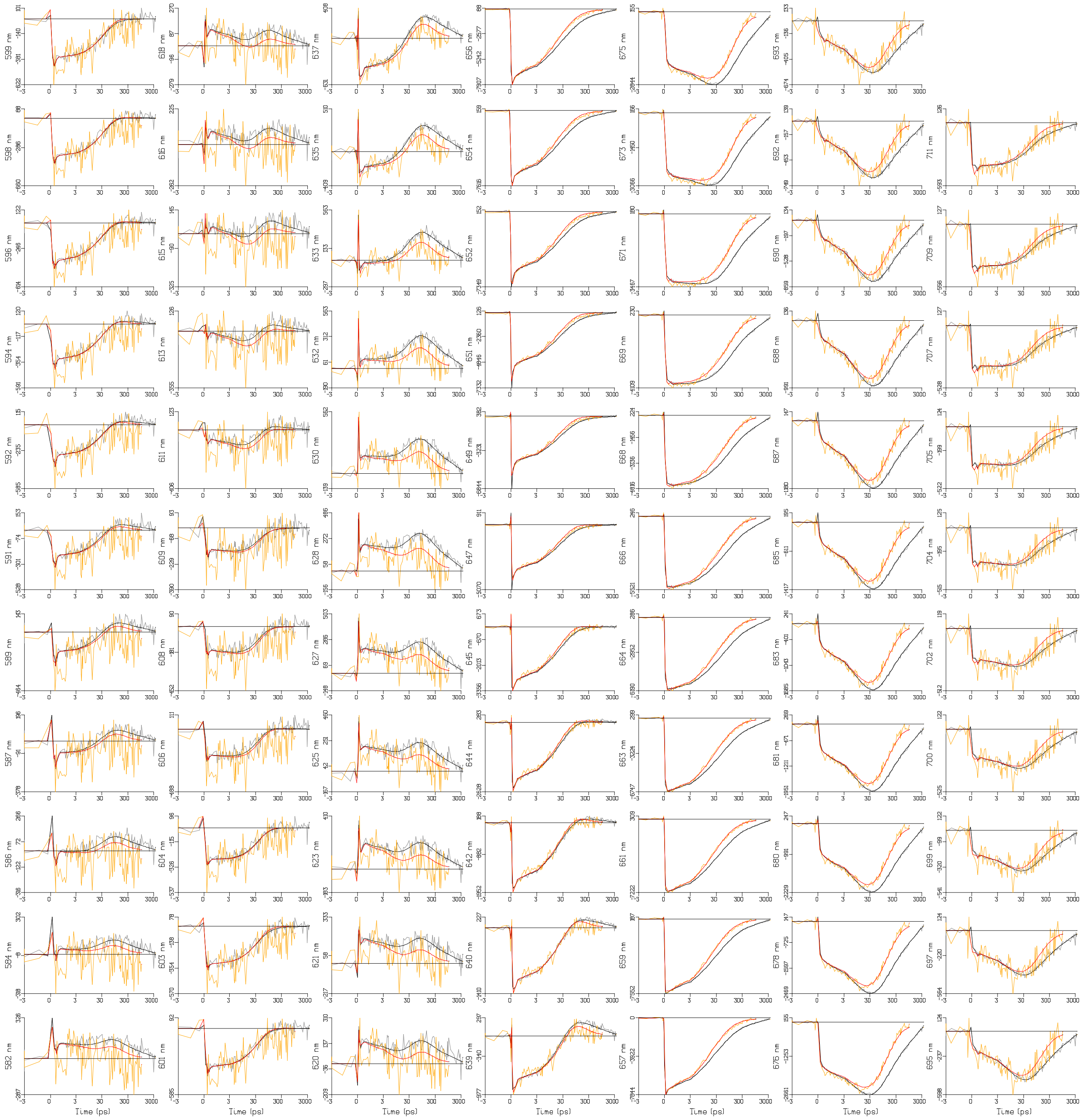

Figure S 11. Transient absorption (in units of µOD) of CB PB at 76 wavelengths (indicated in the abscissa label) after 650 nm excitation of 5 nJ. Key: unquenched (grey), quenched (orange). Black and red lines indicate the simultaneous target analysis fit. Note that the time axis is linear until 3 ps and logarithmic thereafter. Note also that each panel is scaled to its maximum. Overall rms error of the fit was 95 µOD.

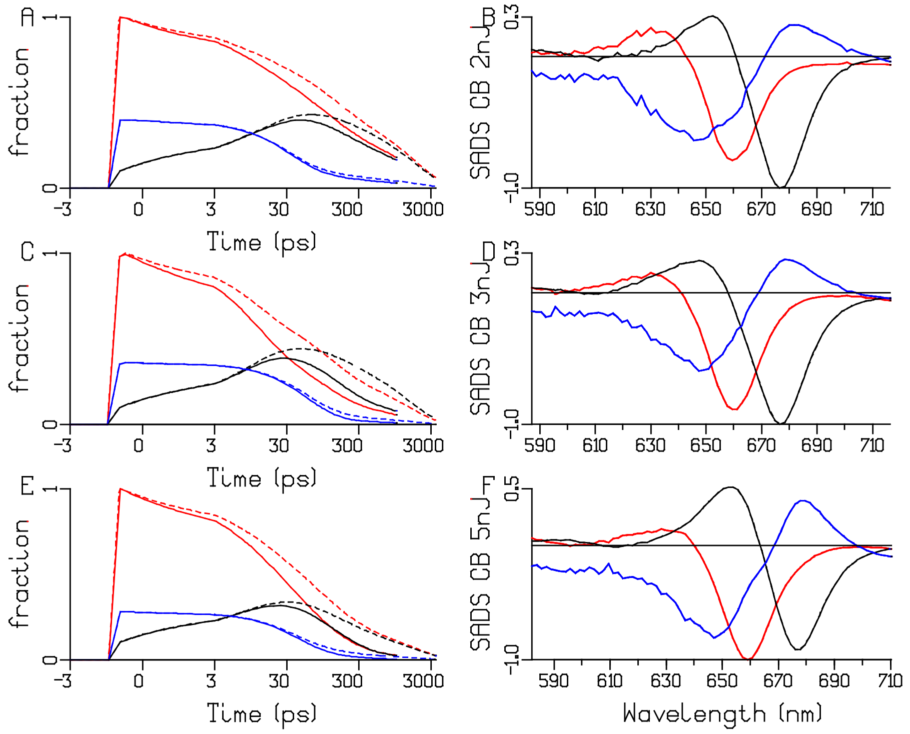

Figure S 12. Total concentrations of APC660 (red), APC680 (black) and rod (blue) and estimated SADS with CB PB. Laser power is indicated in the ordinate label of the SADS. Key: unquenched (dashed) and quenched (solid). Note that the time axis is linear until 3 ps and logarithmic thereafter.

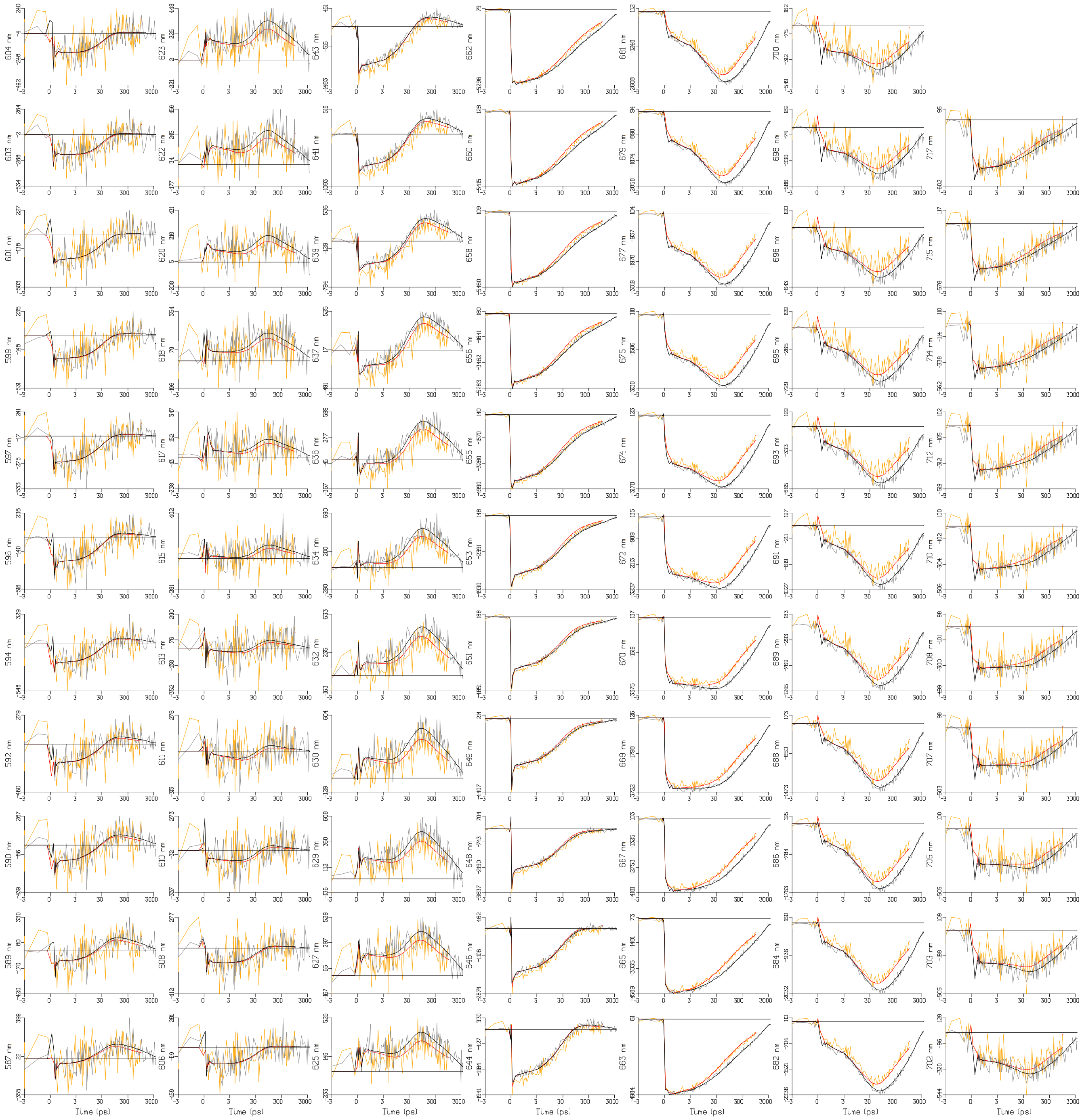

Figure S 13. Transient absorption (in units of µOD) of CB PB at 76 wavelengths (indicated in the abscissa label) after 650 nm excitation of 2 nJ. Key: unquenched (grey), quenched (orange). Black and red lines indicate the simultaneous target analysis fit. Note that the time axis is linear until 3 ps and logarithmic thereafter. Note also that each panel is scaled to its maximum. Overall rms error of the fit was 72 µOD.

Unquenched phycobilisome at 3 different powers (WT PB)

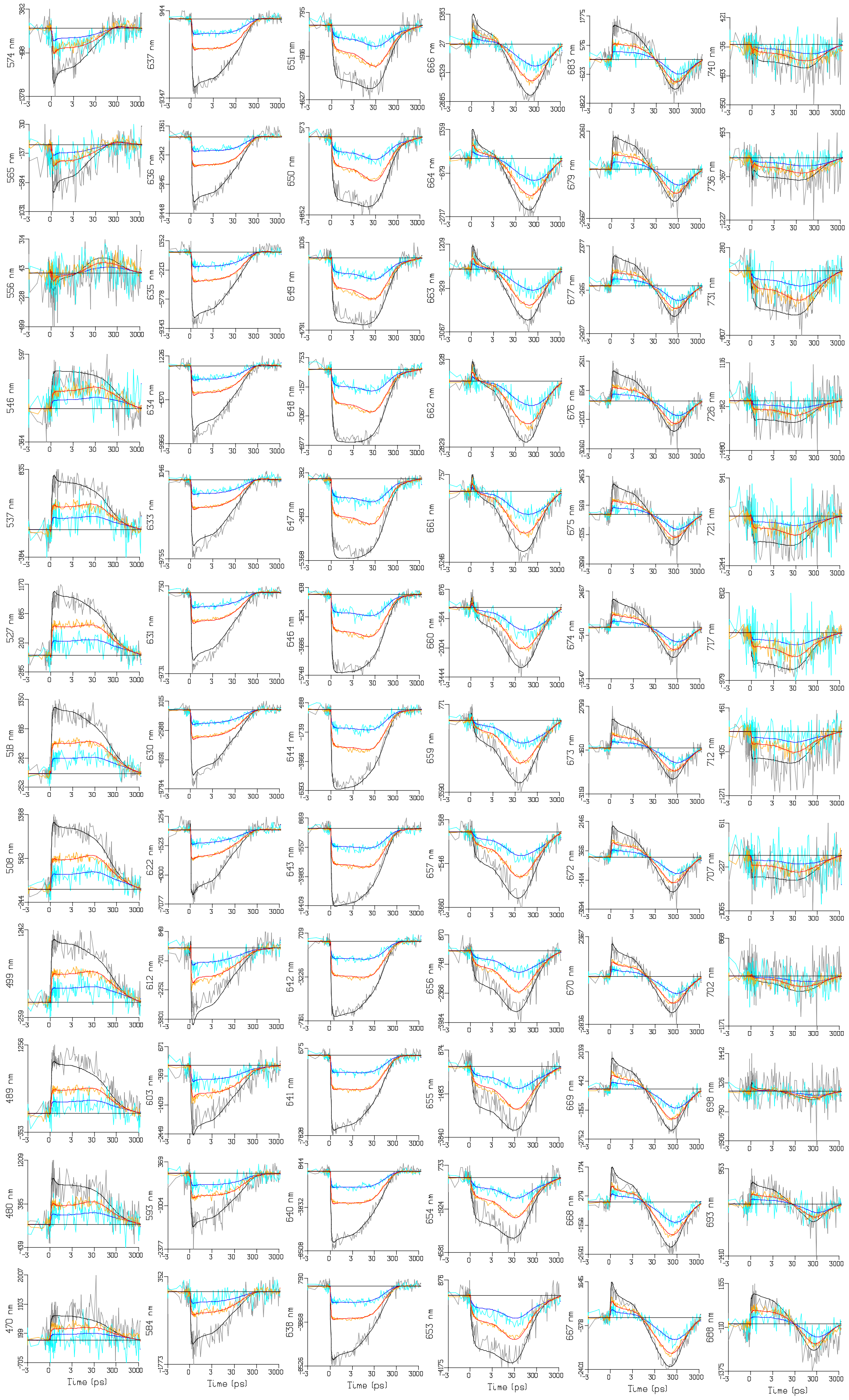

Figure S 14. Transient absorption (in units of µOD) of WT PB at 72 representative wavelengths (indicated in the abscissa label) after 622 nm excitation. Key: 10 nJ (grey), 5 nJ (orange), 2 nJ (cyan). Black, red and blue lines indicate the simultaneous target analysis fit. Note that the time axis is linear until 3 ps and logarithmic thereafter. Note also that each panel is scaled to its maximum. Overall rms error of the fit was 138 µOD.

Unquenched phycobilisome core at 3 different powers (CK PB)

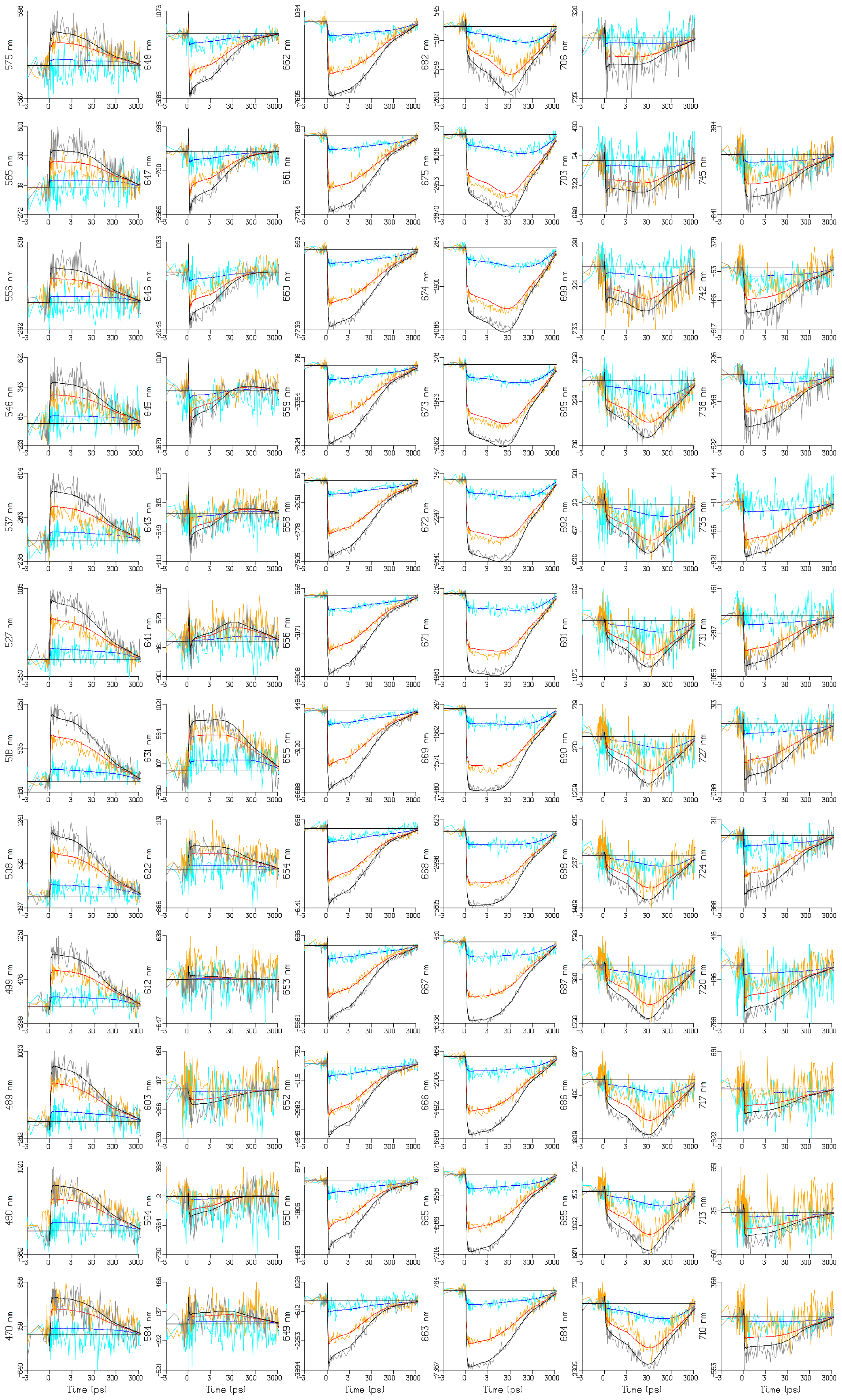

Figure S 15. Transient absorption (in units of μOD) of CK PB at 72 representative wavelengths (indicated in the abscissa label) after 656 nm excitation. Key: 12 nJ (grey), 8 nJ (orange), 2 nJ (cyan). Black, red and blue lines indicate the simultaneous target analysis fit. Note that the time axis is linear until 3 ps and logarithmic thereafter. Note also that each panel is scaled to its maximum. Overall rms error of the fit was 158 μOD.

Time-resolved Emission spectroscopy: Target analysis results

Quenched and unquenched phycobilisome (WT PB)

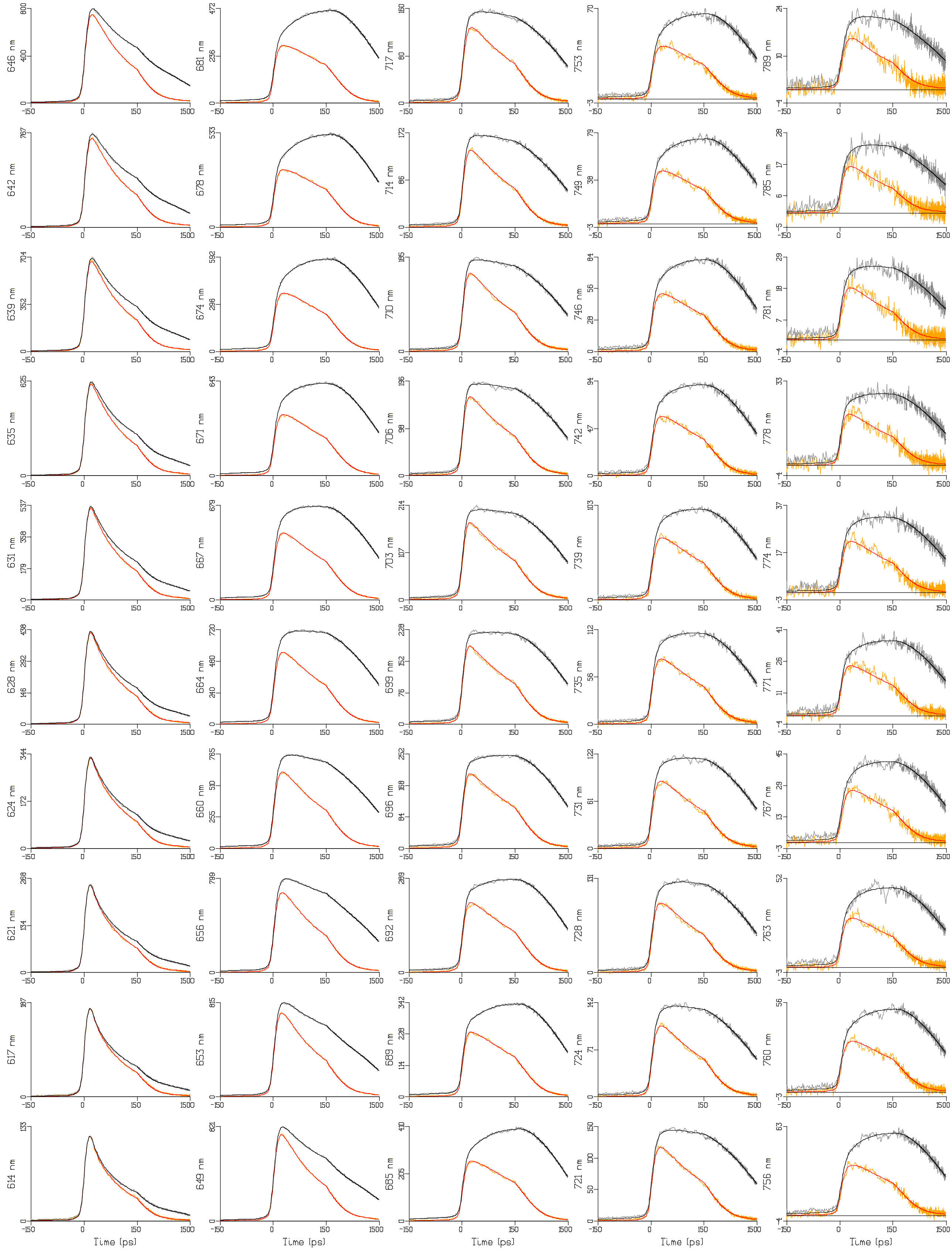

Figure S 16. Emission of WT PB at 50 wavelengths (indicated in the ordinate label) after 590 nm excitation. Key: unquenched (grey), quenched (orange). Black and red lines indicate the simultaneous target analysis fit. Note that the time axis is linear until 150 ps and logarithmic thereafter. Note also that each panel is scaled to its maximum. Overall rms error of the fit was 3.35.

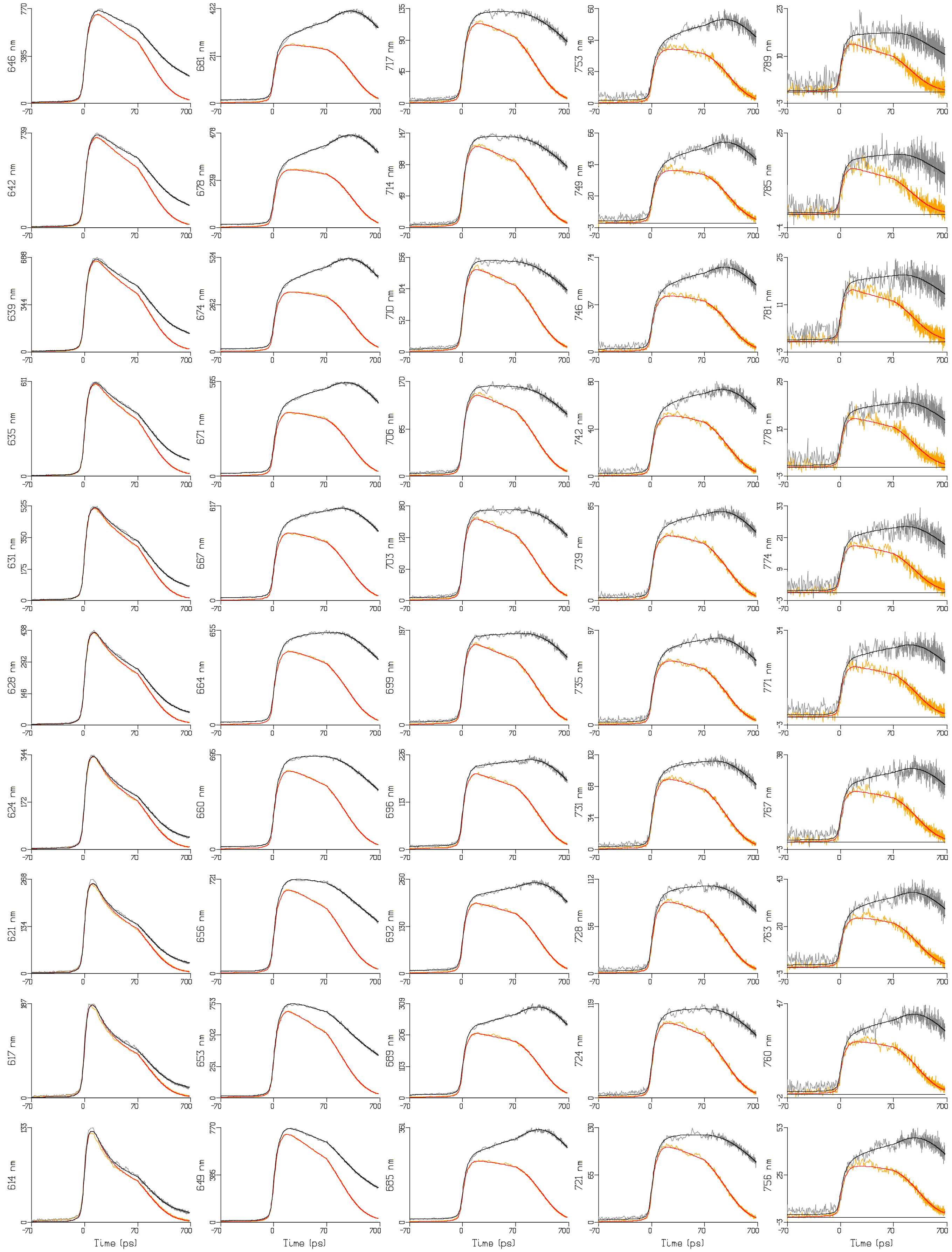

Figure S 17. Emission of WT PB with better time resolution at 50 wavelengths (indicated in the ordinate label) after 590 nm excitation. Key: unquenched (grey), quenched (orange). Black and red lines indicate the simultaneous target analysis fit. Note that the time axis is linear until 70 ps and logarithmic thereafter. Note also that each panel is scaled to its maximum. Overall rms error of the fit was 2.639.

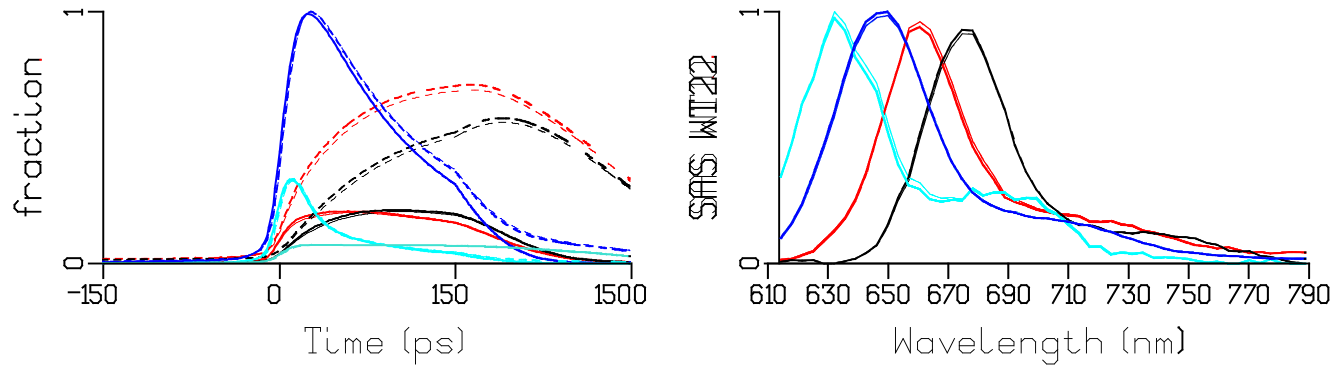

Figure S 18. Overlay of total concentrations and SAS estimated from TRES after 590 nm exc of WT PB with kinetic scheme WT22, Figure 7 (thick lines), and kinetic scheme WT10, Figure 8 (thin lines). Key: PC640 (cyan), PC650 (blue), APC660 (red) and APC680 (black), unquenched (dashed), quenched (solid). Turquoise indicates the fraction of free rods, which possess the PC650 SAS.

Quenched and unquenched phycobilisome core with short rods (CB PB)

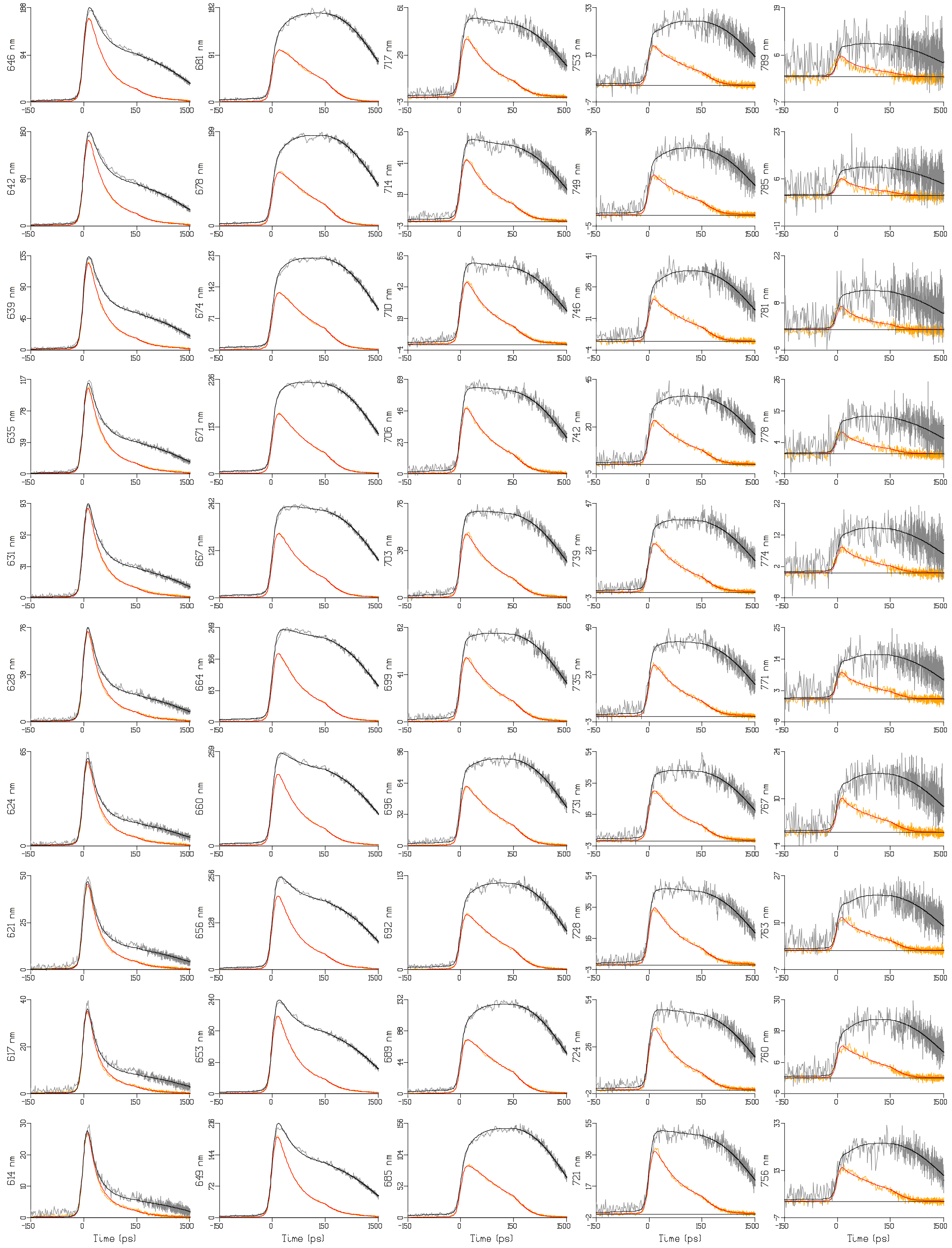

Figure S 19. Emission of CB PB at 50 wavelengths (indicated in the ordinate label) after 590 nm excitation. Key: unquenched (grey), quenched (orange). Black and red lines indicate the simultaneous target analysis fit. Note that the time axis is linear until 150 ps and logarithmic thereafter. Note also that each panel is scaled to its maximum. Overall rms error of the fit was 2.129.

Quenched and unquenched phycobilisome core (CK PB)

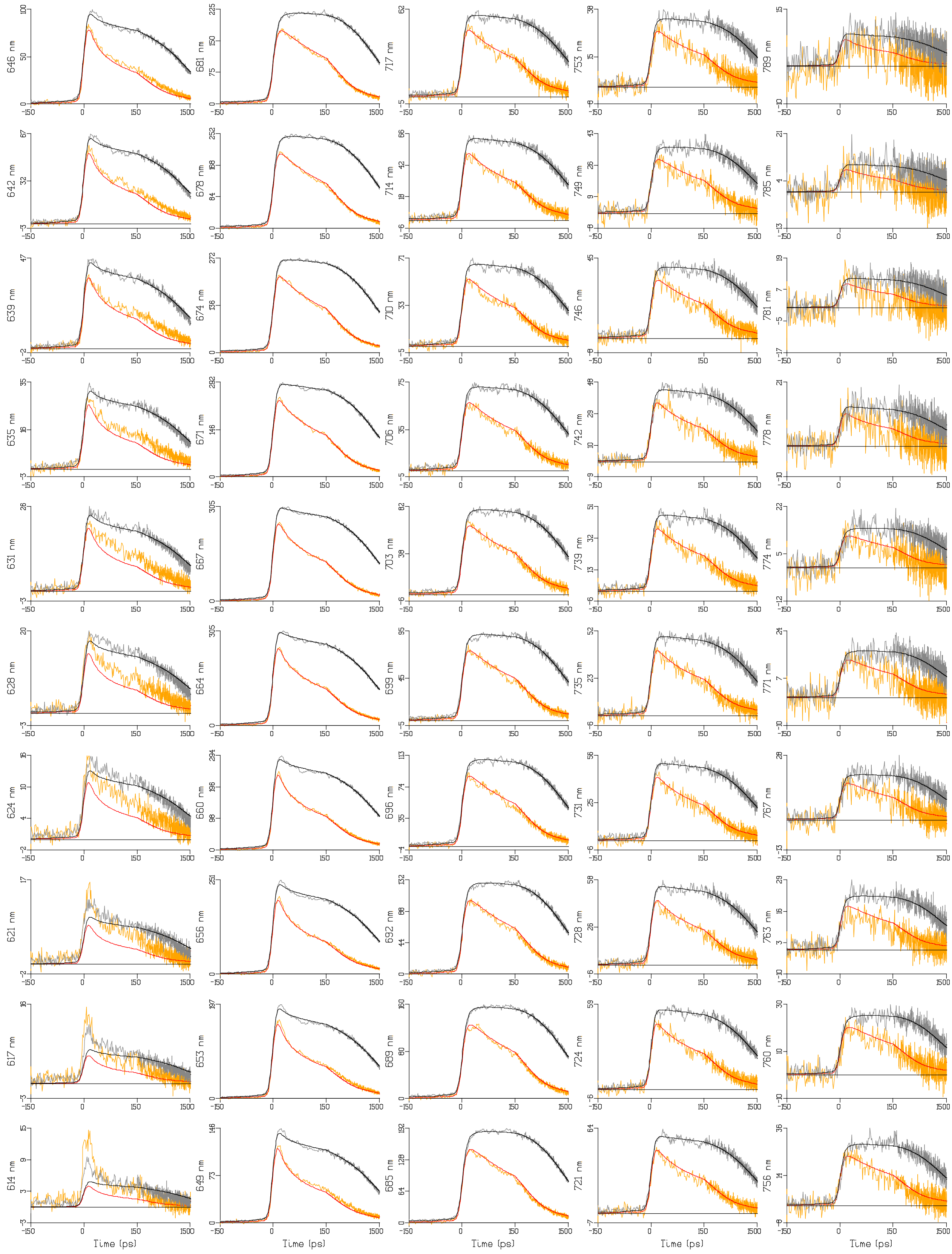

Figure S 20. Emission of CK PB at 50 wavelengths (indicated in the abscissa label) after 590 nm excitation. Key: unquenched (grey), quenched (orange). Black and red lines indicate the simultaneous target analysis fit. Note that the time axis is linear until 150 ps and logarithmic thereafter. Note also that each panel is scaled to its maximum. Overall rms error of the fit was 1.826.

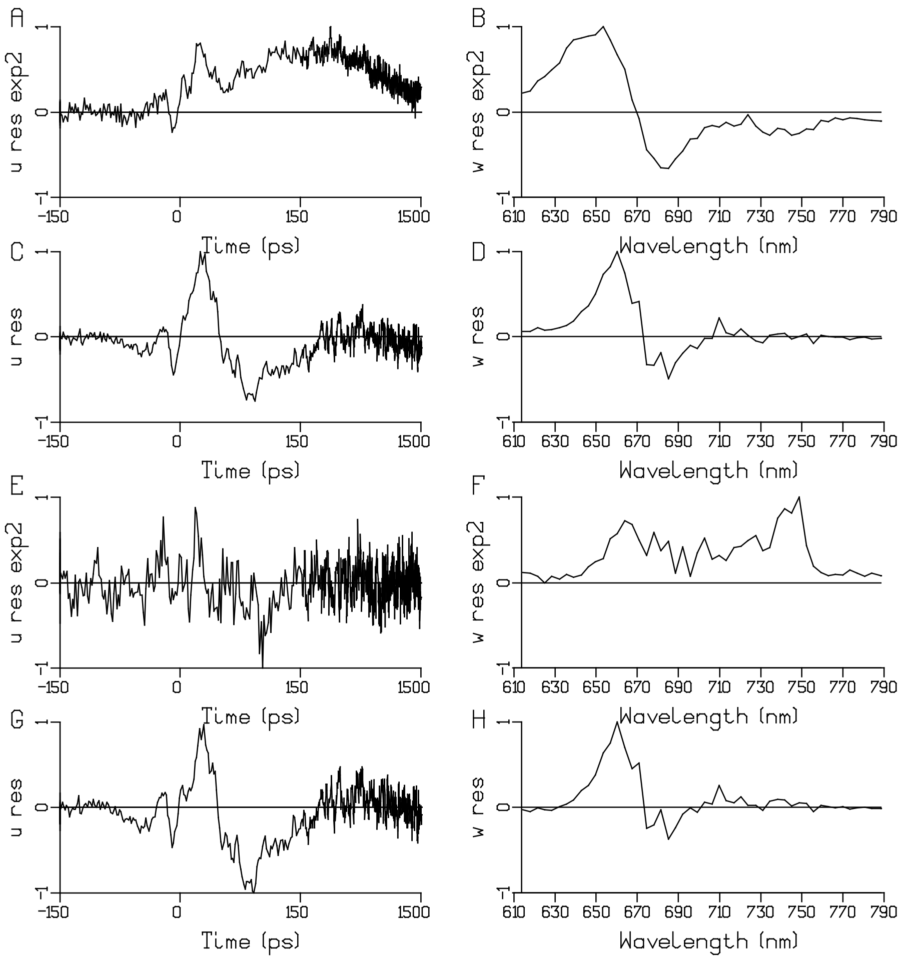

Figure S 21. First left and right singular vectors u_1 (left column) and w_1 (right column) of the residual matrix after a target analysis fit of the TRES data of CK PB in the unquenched (U) and quenched (Q) state. (A,B) Q data, and (C,D) U data, kinetic scheme CK4 (Figure 12A). (E,F) Q data, and (G,H) U data, kinetic scheme CK6 (Figure 12B).

Energy quenching in the CB PB emission revisited

We also performed a target analysis of CB PB with a model that contained additional Q compartments. The rms error of the fit improved significantly, from 2.129 to 2.070. The SVD analysis of the residual matrix of the quenched data that results from the target analysis using the CB10 kinetic scheme (Figure S 6) reveals a large improvement when the CB12 kinetic scheme (Figure S 23) is used instead. The structure that is present with CB10, cf. Figure S 22 A,B, has completely disappeared with the CB12 kinetic scheme, cf. Figure S 22 E,F. The estimated Q SAS is practically invisible in Figure S 24B, with oscillator strength hundred times smaller than APC660. But its shape seems reasonable, cf. the normalized green SAS in Figure S 24C.

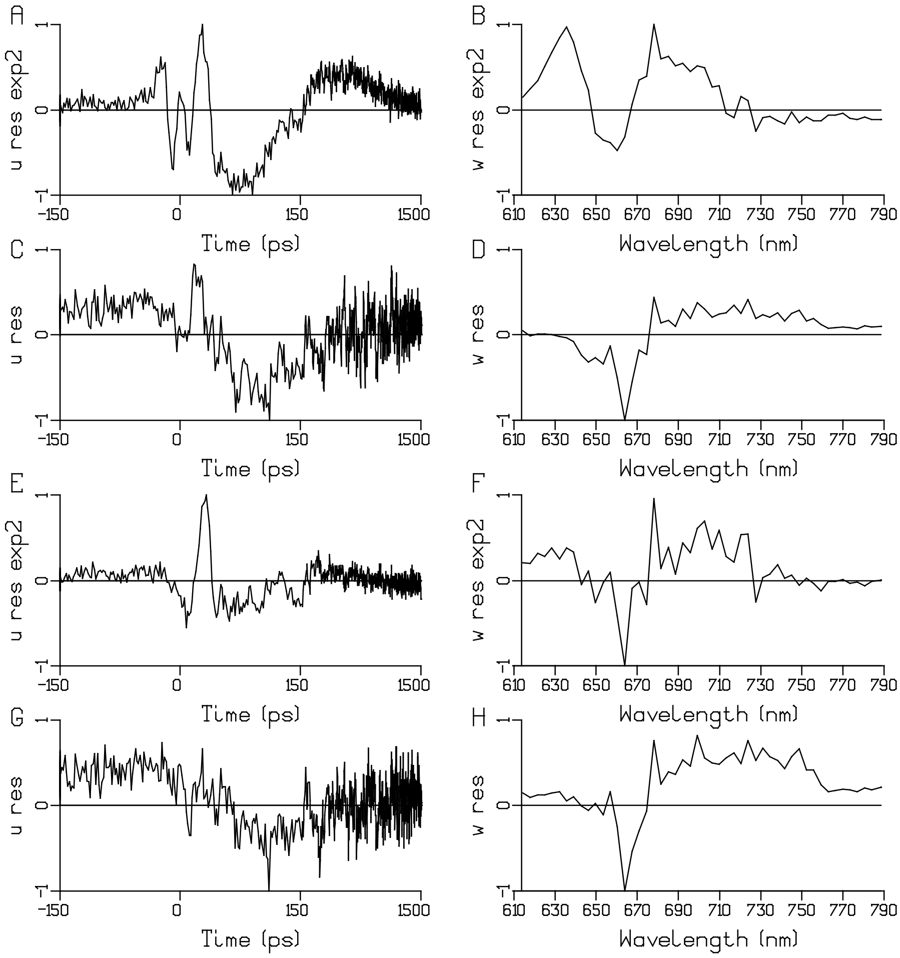

Figure S 22. First left and right singular vectors u_1 (left column) and w_1 (right column) of the residual matrix after a target analysis fit of the TRES data of CB PB in the unquenched (U) and quenched (Q) state. (A,B) Q data, and (C,D) U data, kinetic scheme CB10 (Figure S 6). (E,F) Q data, and (G,H) U data, kinetic scheme CB12 (Figure S 23).

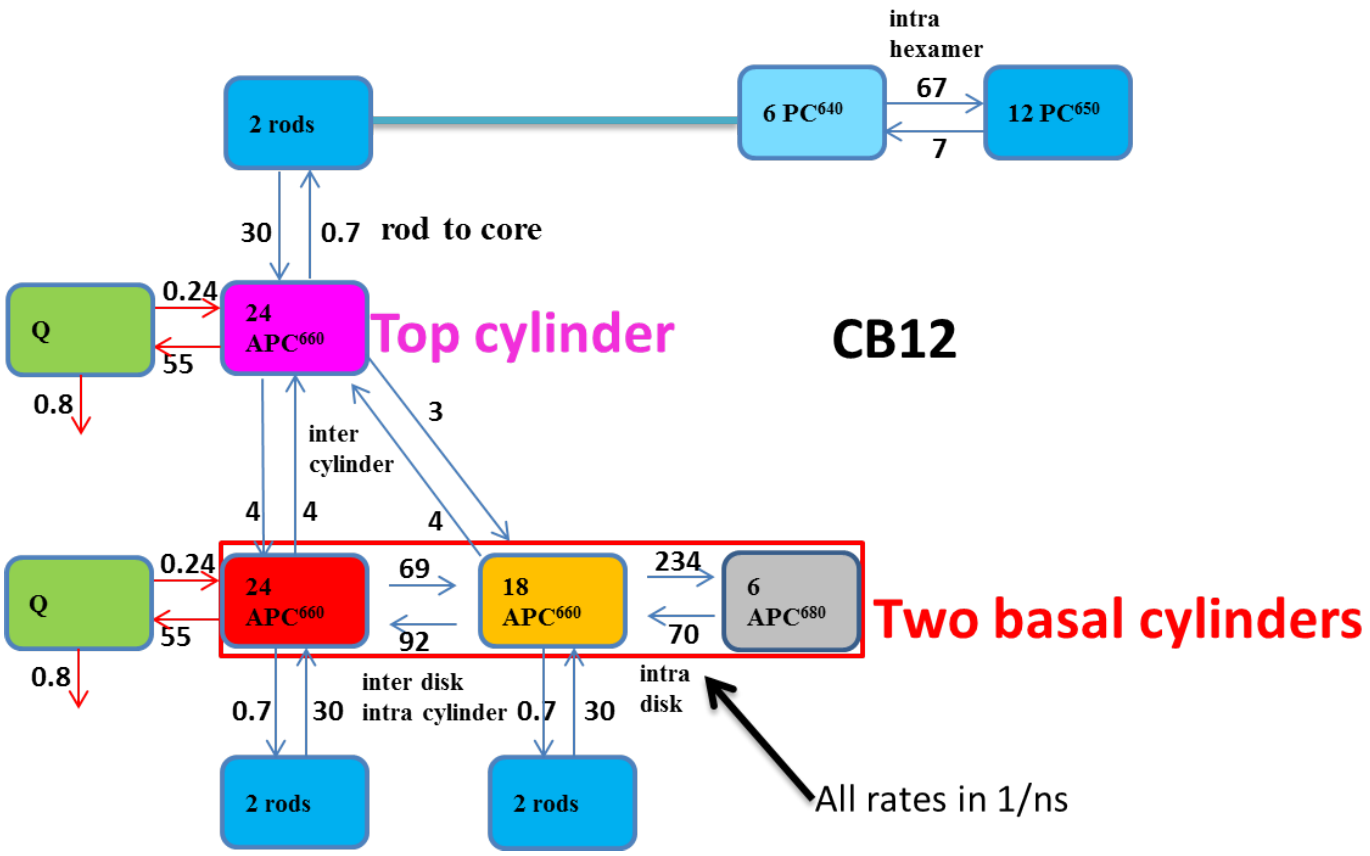

Figure S 23. Kinetic scheme CB12 for energy quenching in CB PB. The common k_{fl} rate constant of 0.6/ns for all excited states except Q has been omitted for clarity.

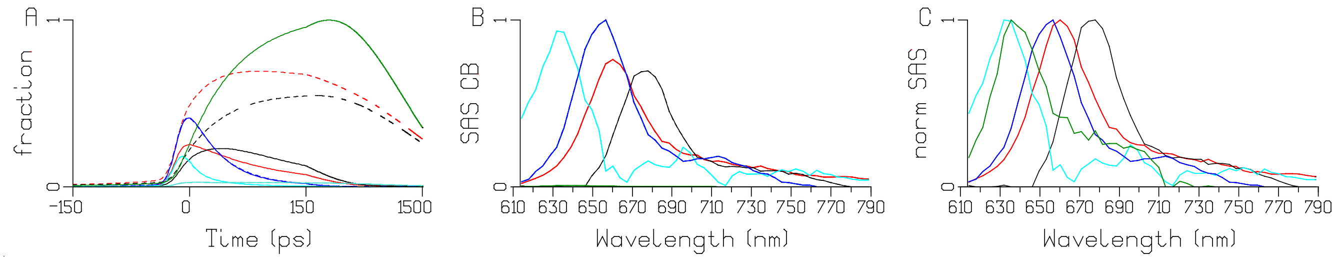

Figure S 24. Total concentrations (A), SAS (B) and normalized SAS (C) estimated from TRES after 590 nm excitation of CB PB using the kinetic scheme CB12 (Figure S 23). Key: Q (green), PC640 (cyan), PC650 (blue), APC660 (red) and APC680 (black), unquenched (dashed), quenched (solid). Turquoise indicates the fraction of free rods, which possess the PC650 SAS.

Energy quenching in the WT PB emission revisited

Finally, we also performed a target analysis of WT PB with a model that contained additional Q compartments. The rms error of the fit improved by 1%, from 2.639 to 2.609. The SVD analysis of the residual matrix of the quenched data that results from the target analysis using the WT10 kinetic scheme (Figure 8) reveals a large improvement when the WT12 kinetic scheme (Figure S 26) is used instead. The structure that is present with WT10, cf. Figure S 25A,B, has almost disappeared at times after 150 ps with the WT12 kinetic scheme, cf. Figure S 25E,F.

Again, the estimated Q SAS is practically invisible in Figure S 27B, with oscillator strength hundred times smaller than APC660. But its shape seems reasonable, cf. the normalized green SAS in Figure S 27C.

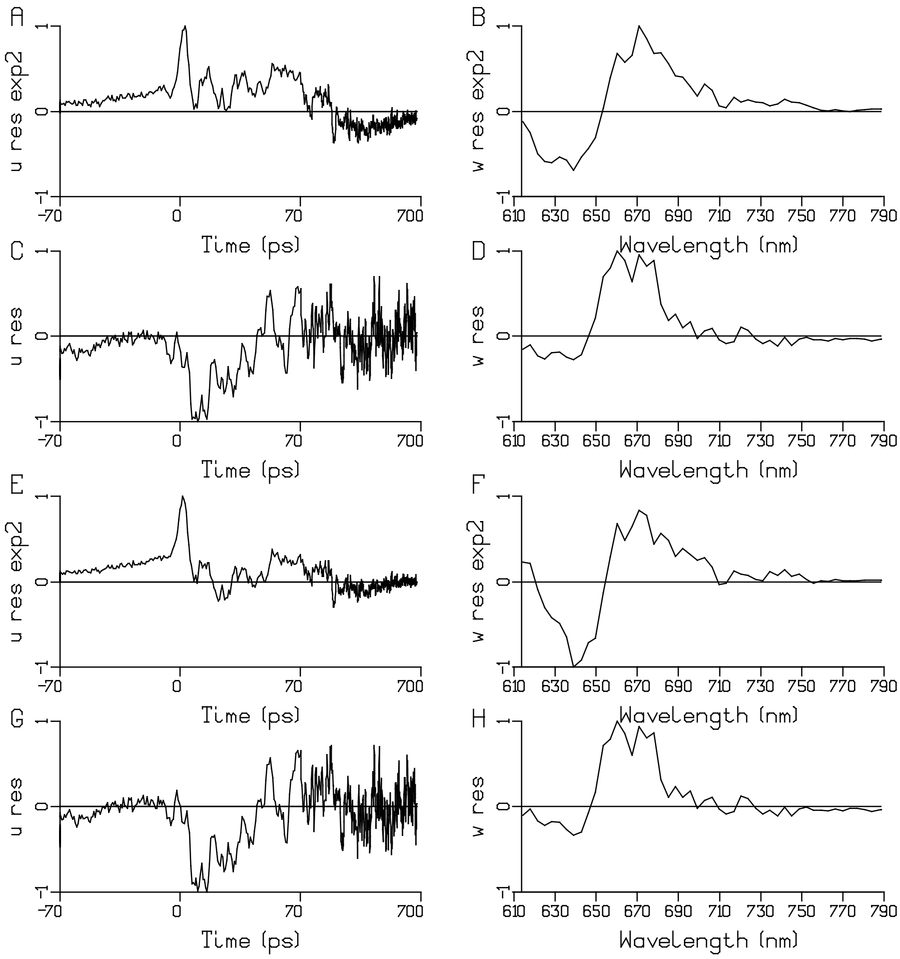

Figure S 25. First left and right singular vectors u_1 (left column) and w_1 (right column) of the residual matrix after a target analysis fit of the TRES data of WT PB in the unquenched (U) and quenched (Q) state. (A,B) Q data, and (C,D) U data, kinetic scheme WT10 (Figure 8). (E,F) Q data, and (G,H) U data, kinetic scheme WT12 (Figure S 26).

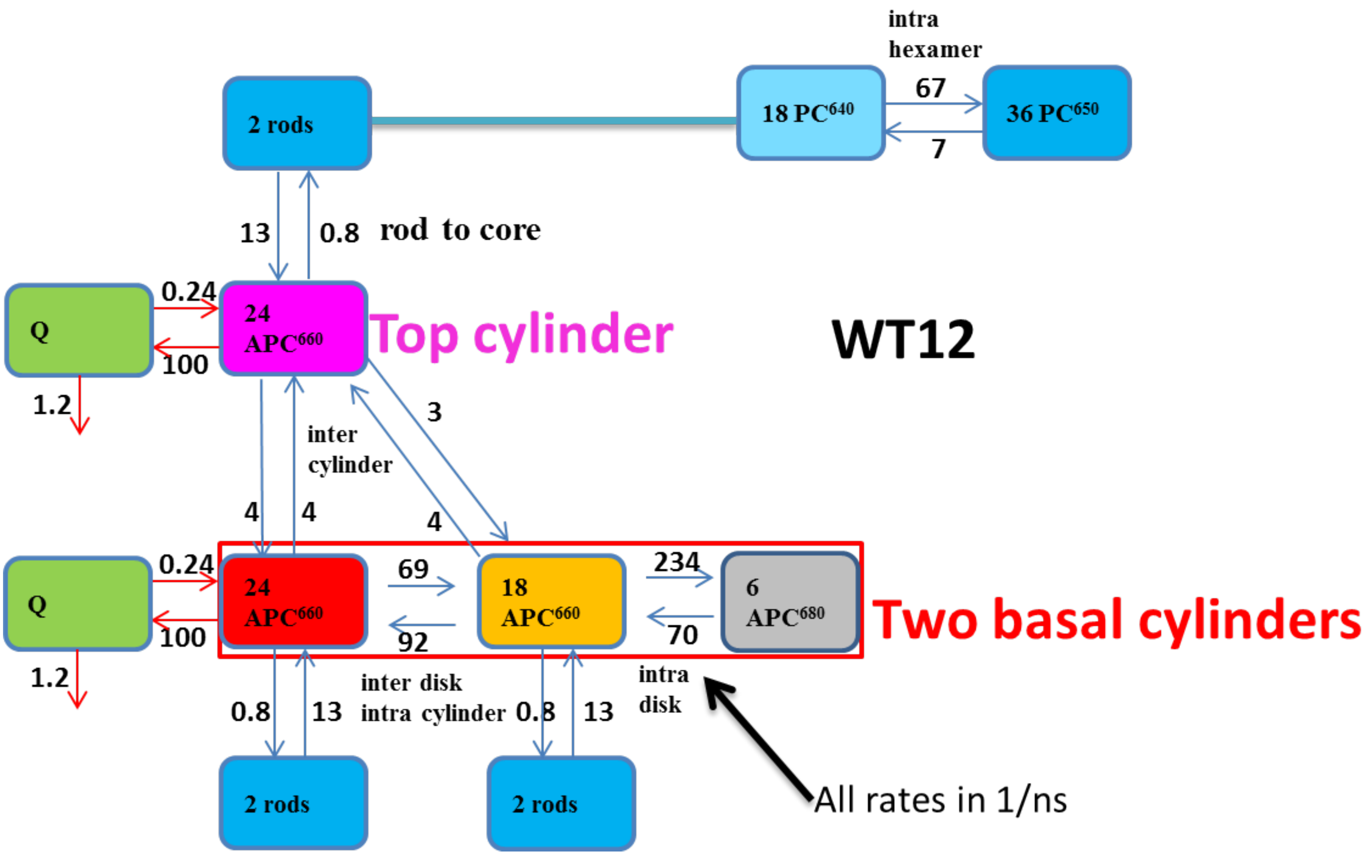

Figure S 26. Kinetic scheme WT12 for energy quenching in WT PB. Depicted here is the scheme for the largest fraction (71%) that is quenched with $k_q = 100/ns$. The common k_{fl} rate constant of 0.6/ns for all excited states except Q has been omitted for clarity.

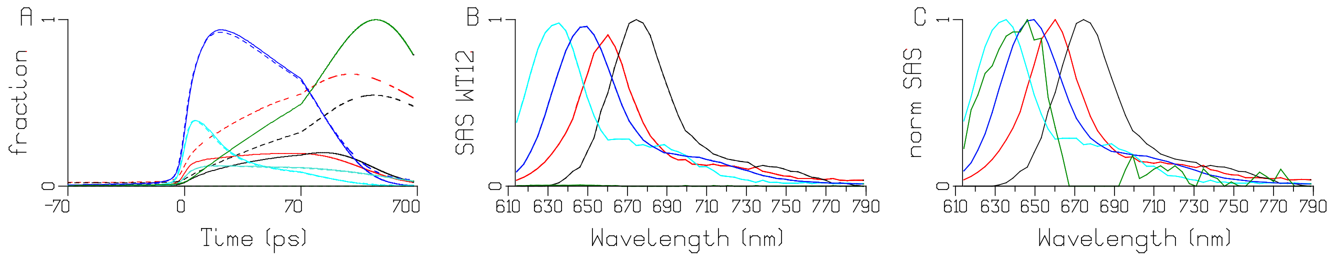

Figure S 27. Total concentrations (A), SAS (B) and normalized SAS (C) estimated from TRES after 590 nm excitation of WT PB using the kinetic scheme WT12 (Figure S 26). Key: Q (green), PC640 (cyan), PC650 (blue), APC660 (red) and APC680 (black), unquenched (dashed), quenched (solid). Turquoise indicates the fraction of free rods, which possess the PC650 SAS.

References

Reuter W, Wiegand G, Huber R, Than ME (1999) Structural analysis at 2.2 Å of orthorhombic crystals presents the asymmetry of the allophycocyanin–linker complex, AP·LC7.8, from phycobilisomes of Mastigocladus laminosus. Proceedings of the National Academy of Sciences 96 (4):1363-1368.
